# Mitochondrial DNA mediates immunoparalysis of dendritic cells in sepsis via STING signalling

**DOI:** 10.1111/cpr.13328

**Published:** 2022-09-15

**Authors:** Qing Tu, Yi Li, Jiali Zhu, Long Guo, Chenchen Liu, Lu Liu, Yuan Yuan, Yun Zou, Feng Chen, Liangfang Yao, Jinbao Li

**Affiliations:** ^1^ Department of Anesthesiology Shanghai General Hospital, Shanghai Jiao Tong University School of Medicine Shanghai China; ^2^ School of Anesthesiology Weifang Medical University Weifang China

## Abstract

**Background:**

Mitochondrial DNA (mtDNA) is a potent activator for pro‐inflammatory response. Dendritic cells (DCs) are immunosuppressed in sepsis, whether mtDNA mediates immunoparalysis in sepsis remains unknown.

**Methods:**

The mRNAs were assessed by qPCR. Flow cytometry was used to measure the expression of costimulatory molecules and the proliferation of CD4^+^ T cells. Western blot and immunofluorescence staining were used to analyse the expression of proteins. Cytokine secretion was detected by ELISA. Histology of lung tissue was used to assess the inflammatory injury.

**Results:**

Lipopolysaccharide‐induced endotoxemia increased plasma mtDNA levels and immunoparalysis of spleen DCs, while hydrolysing mtDNA reversed immunoparalysis of spleen DCs in vivo. Moreover, cytoplasmic mtDNA of DCs was accumulated in endotoxemia and sepsis. mtDNA transfection into bone marrow‐derived DCs (BMDCs) inhibited the expression of costimulatory molecules (e.g., CD40, CD80 and CD86) and the release of IL‐12p70, while increasing the secretion of IL‐10. Cytoplasmic mtDNA also inhibited the ability of BMDCs to promote the proliferation of CD4^+^ T cells. Mechanistic analysis revealed that STING signalling was required for mtDNA‐mediated immunoparalysis of DCs in vivo and in vitro. Further studies showed deletion of STING reversed mtDNA‐mediated immunoparalysis of DCs and improved the prognosis of endotoxemia and sepsis.

**Conclusion:**

Our results demonstrated that mtDNA promotes immunoparalysis of DCs, and contributes to sepsis‐associated immunosuppression by activating STING signalling. Our study may provide new insights to elucidate the molecular pathogenesis of immunosuppressive DCs in sepsis.

## Graphical abstract

Sepsis led to mtDNA accumulation in cytoplasm of DCs. Cytoplasmic mtDNA activated STING signaling to up‐regulate phosphorylation of TBK1, promote the phosphorylation and nuclear translocation of IRF3 to mediate transcription of type I IFNs, which in then triggered the JAK‐STAT3 signaling to up‐regulate the transcription of IL‐10.

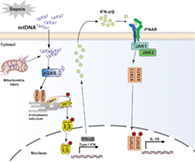

## 1 INTRODUCTION

Sepsis is a life‐threatening disease, with organ dysfunction secondary to the dysregulated host response to severe infection, and with an in‐hospital mortality greater than 10%.^1^ Typically, two phases, ‘hyper inflammation’ and ‘immune paralysis’ phase have been highlighted in the progress of sepsis. Most patients can withstand the hyper inflammation phase and shift to the long‐term immune paralysis stage, which was also described as sepsis‐associated immunosuppression. Susceptibility to opportunistic pathogens increases significantly during ‘immune paralysis’ phase,^2^, ^3^ thus leading to poor prognosis of sepsis. It was suggested that the mortality is up to 85% when patients infected with opportunistic pathogens during immunosuppressive period.^4^ Immune cells paralysis (including monocytes, macrophages, dendritic cells [DCs], etc.) is one of the most typical characteristics in ‘immune paralysis’ phase. As the most important antigen presenting cell, DCs play an essential role in bridging innate and adaptive immunity, participating in maintaining immune homeostasis. Previous studies have indicated that depletion of DCs seriously affects the prognosis of sepsis, which was regarded as a vital target for sepsis treatment.^5^, ^6^ Sepsis associated hyperinflammation allows accumulation of damage‐associated molecular patterns (DAMPs) in peripheral circulation, including high mobility group protein‐1 (HMGB1),^7^ histones, mitochondrial DNA (mtDNA), and so forth.^8^ DAMPs serve as potent activators of the immune system in initiating and perpetuating non‐infectious inflammation to induce systemic inflammation, leading to organ injury and paralysis of immune cells,^9^, ^10^ thus resulting in poor prognosis of sepsis. Sepsis‐associated tissue injury and destruction lead to the release of mtDNA into circulation. Clinical study suggests that free mtDNA in circulation is related to increased mortality of sepsis.^11^ But the potential mechanisms are still not well known.

DCs play an essential role in recognizing pathogens and regulating immune response and inflammation, which are critical for initiating adaptive immune responses and governing immune tolerance.^12^, ^13^ Overproduction of suppressive factors inhibits the functions of immune cells, thus resulting in immune paralysis in sepsis. A large amount of immunoparalysis of DCs (with decrease in the expression of costimulatory molecules, reduction of the release of pro‐inflammatory cytokines and increase in the release of immunosuppressive factors) developed during sepsis,^3^, ^14^ which in turn promoted the generation of Tregs, as well as inhibited the activation of NK cells, CD4^+^ and CD8^+^ T cells, causing sepsis‐associated immunosuppression.^12^ mtDNA is identified as an endogenous immunostimulatory trigger, as its structure is similar to bacterial CpG‐DNA.^15^ mtDNA suppresses endothelial cell proliferation and promotes inflammatory lung injury induced by internalized bacterial endotoxin lipopolysaccharide (LPS).^16^ Moreover, mtDNA induces Treg suppressive activity, and impairs its ability in maintaining tolerance to self‐antigens, which suggests that mtDNA is involved in immunosuppression.^17^, ^18^ Like macrophages, DCs may also sense mtDNA through signal regulatory protein α signalling (SIRPα) by uptaking it into cytoplasm.^19^ Cyclic GMP–AMP synthase (cGAS) detects both self‐DNA and non‐self‐DNA, delivers it to the second messenger, and induces synthesis of cyclic GMP–AMP (cGAMP) which in turn binds to stimulator of interferon (IFN) genes (STING) to increase the production of type I IFNs.^20^, ^21^ However, whether mtDNA affects the phenotypic changes of DCs, specially in promoting immunoparalysis of DCs in sepsis, is still poorly understood.

Herein, we reported that LPS challenged‐endotoxemia and sepsis induced elevation of plasma mtDNA levels and immunoparalysis of spleen DCs in vivo, while hydrolysing mtDNA by DNase‐I partly reversed immunoparalysis of spleen DCs. In sepsis, mtDNA accumulated in DCs cytoplasm, mainly both by uptaking it from extracellular environment and as a result of its own mitochondria damage. We then directly transfected mtDNA into cytoplasm of bone marrow‐derived DCs (BMDCs) to investigate whether cytoplasmic mtDNA led to immunoparalysis of DCs in vitro. The results suggested that cytoplasmic mtDNA markedly induced immunoparalysis of BMDCs by inhibiting the expression of costimulatory molecules (e.g., CD40, CD80 and CD86) and the release of IL‐12p70, but promoted the secretion of IL‐10, and finally restrained the ability of DCs to promote CD4^+^ T cells proliferation. Cytoplasmic mtDNA significantly triggered STING‐type I IFN signalling to induce type I IFNs generation, which increased the production of regulatory cytokine IL‐10 of BMDCs via IFN‐α/β receptor. However, STING deficiency reversed mtDNA‐mediated immunoparalysis of DCs in vivo and in vitro, and alleviated immunosuppression in endotoxemic mice. The results may provide new insights to elucidate the molecular pathogenesis of sepsis‐mediated immunoparalysis of DCs and guide targets for sepsis therapy.

## 2 MATERIALS AND METHODS

### 2.1 Mice

C57BL/6 background mice aged 6–8 weeks were purchased from Jihui Laboratory Animal Care Co., Ltd (Shanghai, China). STING‐deficient mice (STING^−/−^) were purchased from Shanghai Model Organisms Center, Inc. (Shanghai, China). OT‐II mice were kindly provided by Xiaotao Li Lab (Shanghai Key Laboratory of Regulatory Biology, Institute of Biomedical Sciences, School of Life Sciences, East China Normal University). All the mice were housed under controlled in specific pathogen free grade condition (24 ± 2°C, 40–70% relative humidity, 12‐h light/12‐h dark cycle). Mice were free to get food and water ad libitum. All mice received humane care in compliance with institutional animal care guidelines, and all protocols were conducted following the Animals Use Committee of Shanghai General Hospital, Shanghai Jiao Tong University (No. 2019AW009).

### 2.2 LPS model

Endotoxemia/LPS challenge was used as a model for sepsis.^22^ Age‐ and gender‐matched, 6–8 weeks old wild type and STING^−/−^ mice were intraperitoneally injected with a single dose of LPS (10 mg/kg, diluted in phosphate buffer solution (PBS) 200 μl, Sigma, L2630) to generate a model of endotoxemia, while controlled mice received injected with equal volume of PBS (200 μl). Mice were sacrificed 24 h after LPS challenge for sample collection.

### 2.3 Cecal ligation and perforation

Cecal ligation and perforation (CLP) was applied to the established septic mice according to a previous study.^23^ Briefly, mice were anaesthetized with inhaled sevoflurane (2%–4% mixed with air, 1:1). A longitudinal incision was applied for peritoneal cavity exposure, the caecum was exteriorized and ligated at the distal of the ileocecal valve using a non‐absorbable 7‐0 suture (with about 80% caecum ligated). The distal end of the caecum was then punctured with a 21 G needle. Pressed to allow a small drop of faeces extruded through the puncture to ensure the patency of the puncture. Placed back the caecum into the peritoneal cavity and closed the incision. For mice that received sham CLP, the longitudinal incision was made and the caecum was also exposed, but without ligation and perforation. All mice received a single dose subcutaneous injection of 1 ml saline for resuscitation after surgery. Mice were sacrificed 6–48 h after CLP for sample collection.

### 2.4 Clinical scores

Clinical scores were recorded to assess the status of mice according to a previous study.^24^ Briefly, clinical scores of mice were assessed as indicated (0–18 points): (1) clinical appearance (0–4 points); (2) behaviour change (0–6 points); (3) clinical physiological signs (0–3 points); (4) hydration status (0–5 points). The higher scores indicate the worse clinical situation.

### 2.5 BMDCs generation in vitro

The BMDCs were generated as previously described.^25^ Briefly, bone marrow from 6 weeks old mice was harvested and cultured in RPMI‐1640 medium (Gibco) supplemented with 10% foetal bovine serum (Gibco), recombinant mouse GM‐CSF (10 ng/ml, PeproTech, AF‐315‐03‐20) and Murine IL‐4 (1 ng/ml, Peprotech, 214‐14‐20). Fresh medium was supplemented on the third and sixth day after culture, and the non‐adherent cells were harvested and used in further experiments on the seventh day.

### 2.6 Cytokine secretion assays in vivo and in vitro

Mouse IL‐12p70 Quantikine ELISA Kit (M1270), Mouse IL‐10 Quantikine ELISA Kit (M1000B), Mouse IFN‐beta Quantikine ELISA Kit (MIFNB0) and Mouse IFN‐alpha ELISA Kit (42120‐1), Human IL‐10 Quantikine ELISA Kit (D1000B), Human IL‐12 p70 Quantikine ELISA Kit (D1200) were purchased from R&D Systems. Mouse IL‐6 ELISA Kit (70‐EK206/3‐96) and Mouse TNF‐a ELISA Kit (70‐EK282/3‐96) were purchased from Multisciences (Lianke) Biotech, Co., Ltd. The methods were performed according to the manufacturer's instructions.

### 2.7 Flow cytometry

The following antibodies were used for flow cytometric analyses in this study. APC anti‐mouse CD11c Antibody (117310), PE anti‐mouse CD40 Antibody (124622), PE anti‐mouse CD80 Antibody (104708) and PE anti‐mouse CD86 Antibody (159204) were purchased from Biolegend (San Diego, CA, USA). CD4 Monoclonal Antibody‐FITC (11‐0041‐82) was purchased from eBioscience™ (San Diego, CA, USA).

### 2.8 Histology

After euthanizing with CO_2_ asphyxiation, lungs were harvested and fixed in (vol/vol) 4% paraformaldehyde (PFA) for 24 h, then were embedded in paraffin, serial 4.5‐μm thick sections were prepared and stained with haematoxylin and eosin (H&E) for overall histological analysis. Images were captured with the Lecia DMi8 microscope. The semi‐quantitative scoring system was used to assess the lung injury severity as previously described.^26^

### 2.9 mtDNA isolation and transfection

mtDNA was isolated according to the previous study.^14^ Briefly, mitochondria were purified from wilde‐type mouse liver by using a mitochondrial isolation kit (Beyotime, C3606). The freshly isolated mitochondria were collected and used to isolate mtDNA by using the mtDNA isolation kit (Abcam, Ab65321) according to the manufacturer's instruction. Purified DNA concentration was determined via NanoDrop LITE (Thermo Scientific). The concentration of mtDNA was titrated to 1 μg/μl and immediately stored at −80°C for further use. mtDNA (10 μg/ml) was transfected into cytoplasm of BMDCs via lipofectamine 2000 (Sigma‐Aldrich, L3287‐1ML) according to the previous study.^14^ Then, the total cytoplasmic DNA was isolated by using the DNeasy Blood and Tissue Kit (QIAGEN, 69504), according to the manufacturer's manual. Then quantitative real‐time PCR analysis was performed to relatively quantify the levels of mtDNA, primer sequences are included in Table S1.

### 2.10 Quantitative RT‐PCR analysis

Total RNA was collected and extracted from tissues or cells by using Trizol Reagent (Takara, T9108). RNA concentration and quality were detected (NanoDrop LITE, Thermo Scientific). Reverse transcription was performed with SuperScript®III Kit (Thermo Scientific, 18080093) according to the manufacturer's manual, and the cDNA was subjected to quantitative real‐time PCR. The PCR primers used were provided in Table S1. Data were analysed using the comparative analysis of relative expression based on ^ΔΔ^CT methods.

### 2.11 Immunoblots

The BMDCs were lysed with 0.5 ml of 1× ice‐cold RIPA lysis buffer (NCM Biotech, WB3100) containing 1× EDTA‐Free Protease Inhibitor Cocktail (NCM Biotech, P001) and 1× Phosphatase Inhibitor Cocktail 2 (Sigma‐Aldrich, P5726). The lysates were centrifuged and protein concentration was quantified by BCA assay kit (Beyotime, P0012S), according to manufacturer's instructions. Equal amount of samples were separated in sodium dodecyl sulfate polyacrylamide gel electrophoresis and transferred onto polyvinylidene difluoride membranes (IPVH00010, Millipore). After blocking with 5% non‐fat milk for 2 h at room temperature, the membranes were incubated at 4°C overnight with primary antibodies as following. SIRP‐α (1:2000, 14482‐1‐AP), GAPDH (1:5000, 60004‐1‐lg) and IL‐10 (1:2000, 20850‐1‐AP) were purchased from ProteinTech (Chicago, USA). p‐TBK1 (1:2000, 5483S), TBK1 (1:2000, 38066S), p‐IRF3 (1:2000, 37829S), IRF3 (1:2000, 11904S), STING (1:2000, 13647S), IRF5 (1:2000, 96527S), Cleaved Caspase‐1 (1:2000, 89332S), Caspase‐1 (1:2000, 24232S), Cleaved‐IL‐1β (1:2000, 63124), precursor IL‐1β (1:2000, 12242), p‐JAK1 (1:2000, 74129S), JAK1 (1:2000, 3331S), p‐JAK2 (1:2000, H3771S), JAK2 (1:2000, 3230S) and p‐STAT3 (Tyr705, 1:2000, 9145S) were purchased from Cell Signaling Technology (Boston, USA). TLR9 (1:2000, DF2970), p‐P65 (1:2000, Affinity, AF2006), P65 (1:2000, AF5006) and p‐IRF5 (1:2000, AF8382) were purchased from Affinity (Melbourne, Australia). NLRP3 (1:2000, ab214185) was purchased from Abcam (Cambridge, UK). STAT3 (1:2000, sc‐8019) was purchased from Santa Cruz (CA, USA). Then washed with tris‐buffered saline and tween 20 and incubated with secondary antibody for 2 h at room temperature. Immunoreactive bands were visualized by a BIO‐RAD ChemiDoc XRS system and densitometric analysis was determined by Image J software.

### 2.12 SYTOX green and TMRE staining

SYTOX green was used to stain and label mtDNA. Briefly, mtDNA was isolated according to previous study,^16^ which was stained with SYTOX green (1:100, Beyotime, C1070M) at room temperature for 30 min in the dark, then the SYTOX green labelled‐mtDNA was transfected into the cytoplasm of BMDCs for 24 h. Images were captured with a confocal laser‐scanning microscope (Lecia, SP8 X). To detect the mitochondrial membrane potential (MMP), BMDCs were treated as indicated and incubated with TMRE dilution (1:1000, Beyotime, C2001S) for 30 min at 37°C in the dark. After washing with PBS, BMDCs were captured with the fluorescence microscope (Lecia, DMi8).

### 2.13 Immunofluorescence staining

BMDCs were seeded onto sterile coverslips in culture dishes for indicated treatment. Thereafter, cells were washed twice with PBS, and fixed with 4% PFA for 10 min, then permeabilized with Triton X‐100 (0.5%) for 10 min, and blocked with 1% bovine serum albumin (BSA) for 1 h at room temperature. Then incubated with the primary antibody in 2% BSA overnight at 4°C. After washing with PBS and tween 20, cells were incubated with Cy3‐conjugated anti‐rabbit (Beyotime, A0516) for 1 h at room temperature in the dark. Then fixed coverslips on slides and incubated with DAPI for 10 min. For spleen tissue, cryosections of frozen spleens, 5 μm in thickness, were prepared (Leica®). Sections were fixed with iced acetone and blocked for 1 h with 1% (wt/vol) BSA in PBS. Indicated antigens were stained with the antibodies against mouse antigens CD11c (1:200, Abcam, ab219799) and CD80 (1:500, Abcam, ab134120). Photomicrographs of cells and spleen sections were captured with the fluorescence microscope (Lecia, DMi8).

### 2.14 Bioinformatic analysis and human samples

Since mtDNA has similar structure to CpG (a bacterial DNA fragment). We selected GSE10147 from the gene expression omnibus (GEO) database (https://www.ncbi.nlm.nih.gov/geo/), in which gene expression data was obtained from human plasmacytoid DCs treated with IL‐3 (an inflammatory cytokine) or IL‐3 plus CpG. MINIML.Limma package (version 3.40.2) of R software was used to identify the differential expression of mRNAs as adjusted *p* < 0.05 and absolute value of fold change >1.5. ClusterProfiler package (version 3.18.0) in R was applied to analyse the gene ontology (GO) functions and the Kyoto Encyclopedia of Genes and Genomes (KEGG) pathways enrichment. All the analysis methods and R package were applied by R foundation for statistical computing (2020) version 4.0.3.

Plasma samples of 21 septic patients (on the seventh day after diagnosis) and 19 healthy participants were collected. Informed consents were obtained from all the individuals or their families. The demographics of the participants are shown in Table S2.

### 2.15 Data analysis

Statistical analysis was performed by GraphPad Prism (version 8.2.0, San Diego, CA). For comparisons between two groups, the Student's *t*‐test (unpaired and paired) was applied. Multiple comparisons were performed by one‐ or two‐way ANOVA. Pearson correlation coefficient was used to analyse the linear correlation of two variables. Survival data were analysed by Log‐rank (Mantel–Cox) test. A *p*‐value <0.05 was considered statistically significant. Data were shown as mean ± SEM.

## 3 RESULTS

### 3.1 The elevation of peripheral mtDNA levels mediated immunoparalysis of splenic DCs in sepsis

**FIGURE 1:**
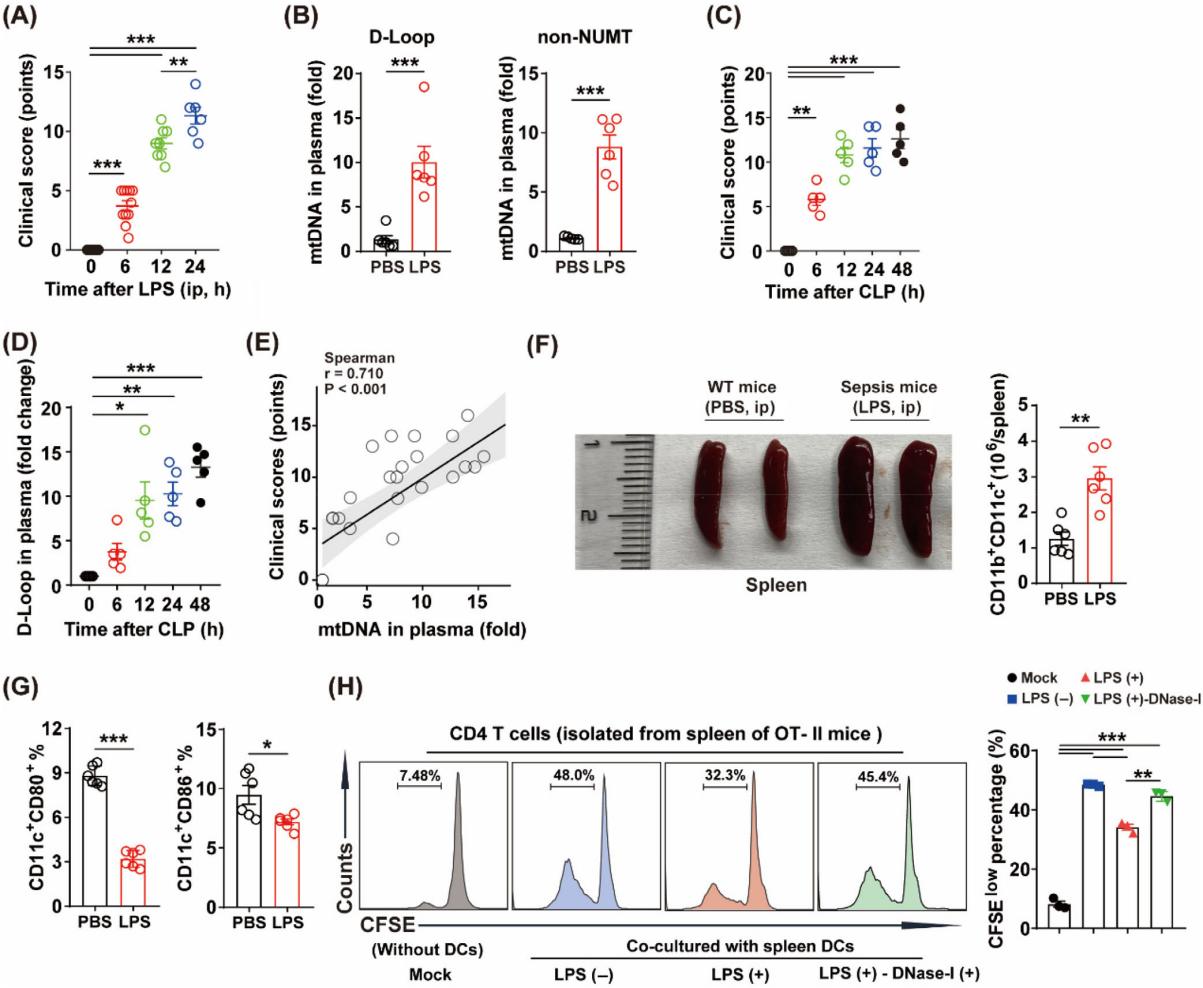
Sepsis associated elevated peripheral mtDNA correlates with disease severity of sepsis and contributes to immunoparalysis of spleen DCs. Intraperitoneal injection of LPS (10 mg/kg) and CLP were applied to established endotoxin mice model. (A) Clinical scores of mice treated with LPS (ip, *n* = 6–12). (B) Peripheral mtDNA levels of mice treated with LPS (ip, *n* = 6) or PBS (ip, *n* = 6). (C) Clinical scores of mice after CLP (*n* = 5). (D) Peripheral mtDNA levels of mice after CLP (*n* = 5). (E) Correlation analysis between peripheral mtDNA and clinical scores. (F) Representative image of spleen and CD11b^+^ CD11c^+^ cells in spleen (*n* = 6). (G) CD11c^+^, CD80^+^ and CD11c^+^ CD86^+^ cells in spleen (*n* = 6). (H) Proliferation of CD4^+^ T cells co‐cultured with spleen DCs (*n* = 3). Data were shown as the mean ± SEM. *p < 0.05; **p < 0.01; ***p < 0.001. CFSE, 5,6‐carboxyfluorescein diacetate succinimidyl ester; CLP, cecal ligation and puncture; DC, dendritic cell; LPS, lipopolysaccharide; mtDNA, mitochondrial DNA.

Sepsis promoted the release of mtDNA into the peripheral circulation.^9^ First, the LPS‐challenge endotoxemia was used as a model for sepsis. Endotoxic mice exhibited poor clinical scores (Figure 1A) and elevated plasma mtDNA (D‐Loop and non‐NUMT) levels (Figure 1B). CLP is used widely in rodents to mimic the physiology of sepsis.^23^ In line with the above results, CLP also increased the clinical scores (Figure 1C), and elevated the level of D‐Loop (Figure 1D) time‐dependently. Correlation analysis indicated that the levels of circulating mtDNA were correlated with clinical scores (Figure 1E), which indicated that circulating mtDNA was associated with the severity of sepsis. In vivo, LPS‐challenge led to markedly splenomegaly with the increased number of splenic DCs (Figure 1F). However, LPS‐challenge inhibited the expression of CD80 and CD86 of spleen DCs (Figure 1G). These data indicated that sepsis could inhibit the expression of costimulatory molecules in DCs in vivo. We further isolated spleen DCs, and co‐cultured with CD4^+^ T cells isolated from the spleen of OT‐II mice and labelled with CFSE (Figure S1). Sepsis restrained the abilities of spleen DCs to promote CD4^+^ T cell proliferation, which was partially counteracted by hydrolysing mtDNA by using DNase I (Figure 1H). These data indicated that elevation of peripheral mtDNA levels resulted in immunoparalysis of spleen DCs in vivo.

### 3.2 Sepsis mediated mtDNA accumulation in cytoplasm of DCs in vivo and in vitro

**FIGURE 2:**
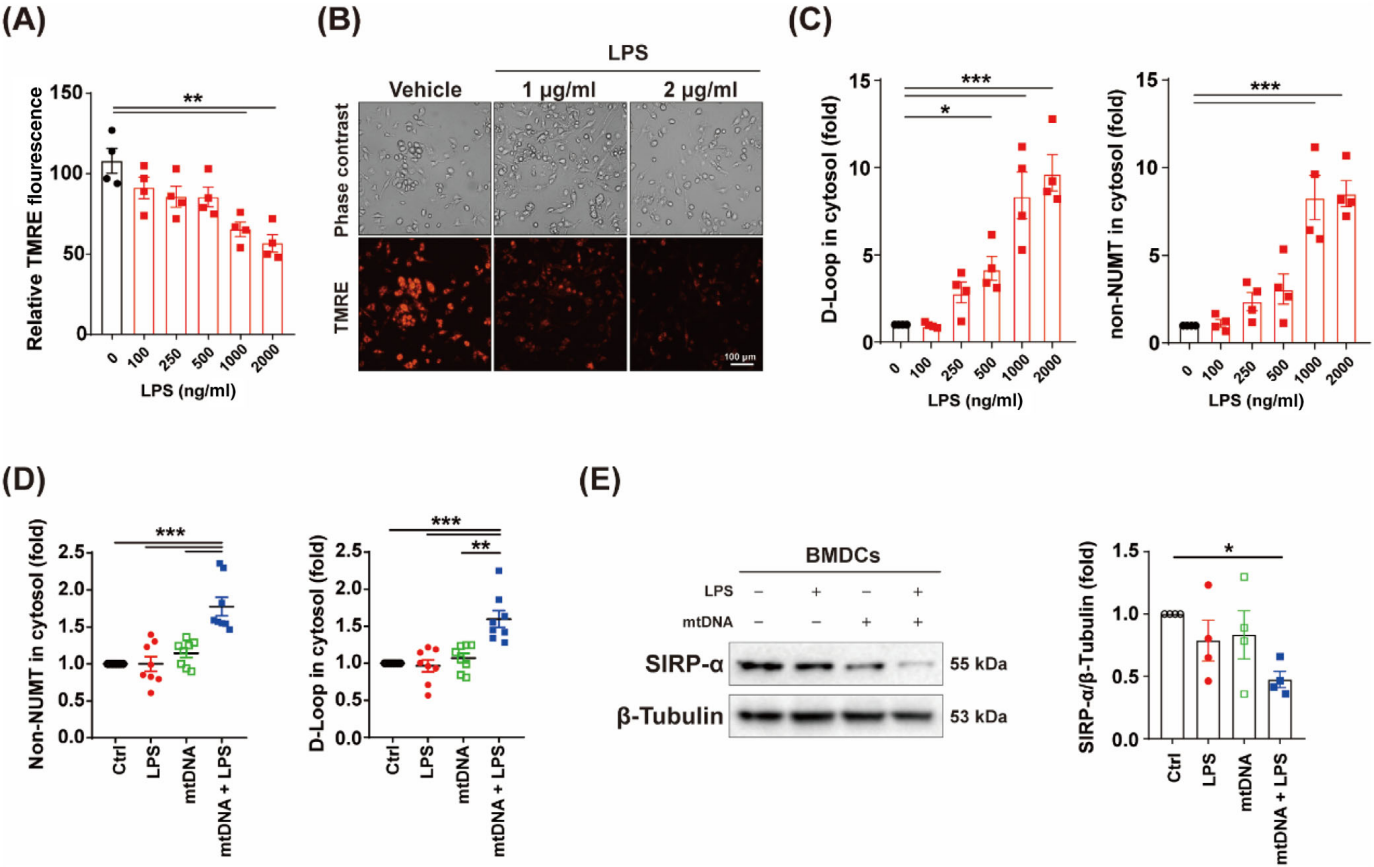
Sepsis induced cytoplasmic mtDNA accumulation. (A) Relative TMRE fluorescence of BMDCs treated with indicated LPS (0, 100, 250, 500, 1000 and 2000 ng/ml) for 24 h (*n* = 4). (B) Representative TMRE image of BMDCs treated with vehicle or LPS (1000 and 2000 ng/ml) for 24 h. Scale bar: 100 μm. (C) Levels of cytoplasmic mtDNA of BMDCs treated as (A) (*n* = 4). (D) Levels of cytoplasmic mtDNA of BMDCs treated with LPS (100 ng/ml) or mtDNA (10 μg/ml) or both combined (*n* = 8). (E) Protein expression and quantification of SIRP‐α BMDCs treated as (D) (*n* = 4). Data were shown as the mean ± SEM. *p < 0.05; **p < 0.01; ***p < 0.001. BMDC, bone marrow derived dendritic cell; LPS, lipopolysaccharide; mtDNA, mitochondrial DNA; SIRP, signal regulatory protein; TMRE, tetramethylrhodamine ethyl ester perchlorate.

Like macrophages, DCs could also uptake mtDNA from extracellular environment.^17^ In this study, spleen DCs were isolated from CLP mice to detect the cytoplasmic mtDNA (D‐Loop and non‐NUMT) levels. Our results suggested that CLP mediated cytoplasmic mtDNA accumulation in spleen DCs (Figure S2). In vitro, BMDCs were treated with various dose of LPS and the MMP was detected by TMRE probe. The results indicated that LPS decreased MMP in a dose‐dependent manner, especially at dose of 1–2 μg/ml (Figure 2A,B). Then, we detected the cytoplasmic mtDNA levels, and found LPS (1–2 μg/ml) treatment elevated cytoplasmic mtDNA levels significantly (Figure 2C). To imitate the pathological status, BMDCs were treated with LPS plus mtDNA in this study. The result indicated that LPS plus mtDNA treatment elevated cytoplasmic mtDNA levels (Figure 2D). CD47‐SIRP‐α axis dictated the fate of ingested DNA in DCs.^17^ Likewise, the treatment of LPS plus mtDNA inhibited expression of SIRP‐α (Figure 2E), which suggested that DCs may engulf mtDNA from extracellular environment.

### 3.3 Cytoplasmic mtDNA mediated immunoparalysis of BMDCs

**FIGURE 3:**
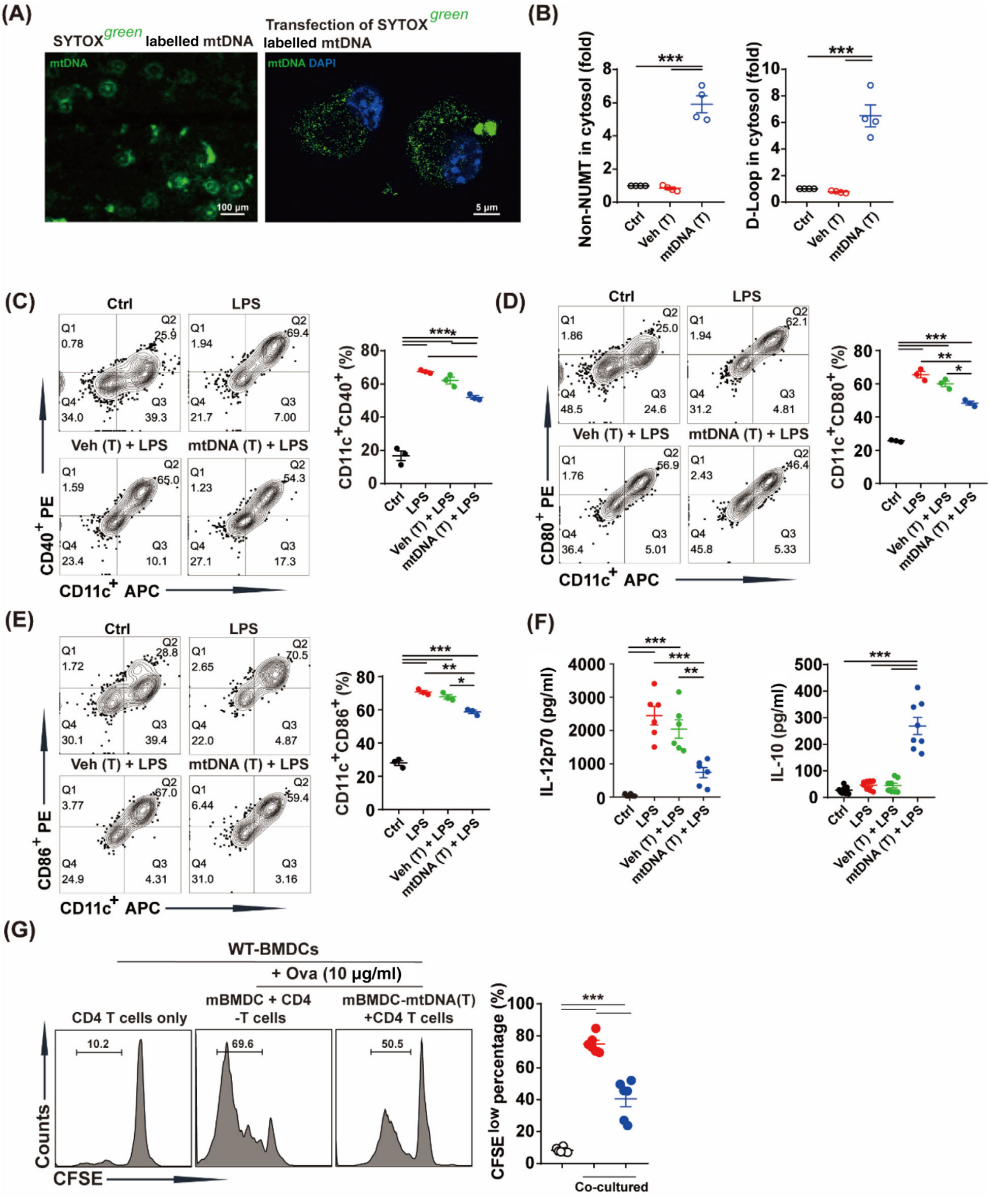
Cytoplasmic mtDNA markedly induced immunoparalysis of BMDCs. (A) mtDNA labelled with SYTOX green and transfection into cytoplasm of BMDCs (green spots). Scale bar: 100 μm (left), 5 μm (right). (B) Levels of cytoplasmic mtDNA of BMDCs transfected with vehicle or mtDNA (10 μg/ml) (*n* = 4). (C) Proportion of CD11c^+^CD40^+^ cells treated with LPS (100 ng/ml) or pretreated with vehicle or mtDNA (10 μg/ml) transfection for 24 h, followed by LPS (100 ng/ml) challenge for 24 h (*n* = 3). (D) Proportion of CD11c^+^CD80^+^ cells treated as (C) (*n* = 3). (E) Proportion of CD11c^+^CD86^+^ cells treated as (C) (*n* = 3). (F) IL‐12p70 and IL‐10 in cultured supernatant of BMDCs treated as (C) (*n* = 6–8). BMDCs were stimulated with LPS (100 ng/ml, 24 h) to activate it (mBMDC) or pretreatment with mtDNA (T) for 24 h and then stimulated with LPS (100 ng/ml, 24 h) to activate it (mBMDC‐mtDNA [T]), then incubated with Ova for 2 h, thereafter co‐cultured with CD4^+^ T cells. (G) Proliferation of CD4^+^ T cells when co‐cultured with BMDCs (*n* = 6). Data were shown as the mean ± SEM. *p < 0.05; **p < 0.01; ***p < 0.001. BMDC, bone marrow derived dendritic cell; CFSE, 5,6‐carboxyfluorescein diacetate succinimidyl ester; LPS, lipopolysaccharide; mtDNA, mitochondrial DNA; mtDNA (T), mtDNA transfection; Ova, ovalbumin; Veh (T), vehicle transfection.

To further investigate whether elevated cytoplasmic mtDNA induced immunoparalysis of DCs in vitro, mtDNA was labelled with SYTOX green, and then transfected into cytoplasm of BMDCs.^16^ Transfection of mtDNA markedly increased the cytoplasmic mtDNA levels (Figure 3A,B). Then, we treated BMDCs with mtDNA transfection followed by LPS challenge. Flow cytometry results indicated that cytoplasmic mtDNA inhibited LPS‐induced expression of costimulatory molecules, including CD40, CD80 and CD86 in BMDCs (Figure 3C–E). Cytoplasmic mtDNA also reduced LPS‐induced production of IL‐12p70, while promoting the secretion of IL‐10 (Figure 3F). Next, mtDNA transfected‐BMDCs were co‐cultured with CD4^+^ T cells. Our results indicated that cytoplasmic mtDNA inhibited the ability of BMDCs to promote the proliferation of CD4^+^ T cells (Figure 3G). These data suggested that cytoplasmic mtDNA mediated immunoparalysis of BMDCs.

### 3.4 Cytoplasmic mtDNA markedly activated STING pathway

**FIGURE 4:**
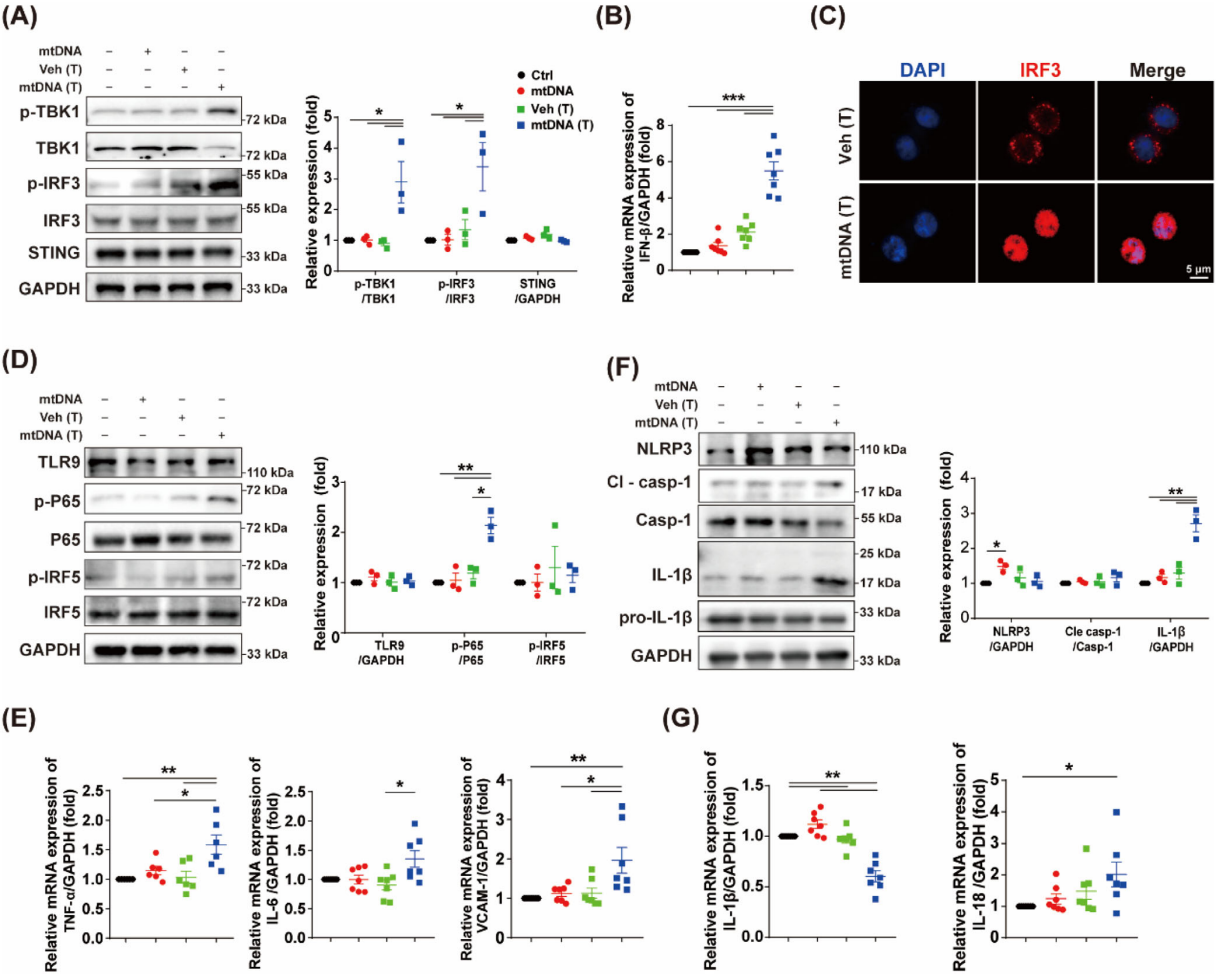
Cytoplasmic mtDNA markedly activated STING signalling. BMDCs were treated with mtDNA (10 μg/ml), or transfected with vehicle or mtDNA (10 μg/ml) for 24 h, then were harvested for western blot and qPCR. (A) Representative blots and relative quantification protein levels of STING pathway (*n* = 3). (B) qPCR quantitation of IFN‐β (*n* = 7). (C) Representative images of nuclear translocation of IRF3 in BMDC treated as indicated. Scale bar: 5 μm. (D) Representative blots and relative quantification protein levels of TLR9 pathway (*n* = 3). (E) qPCR quantitation of TNF‐α, IL‐6 and VCAM‐1 (*n* = 6–7). (F) Representative blots and relative quantification protein levels of NLRP3 pathway (*n* = 3). (G) qPCR quantitation of IL‐1β, IL‐18 (*n* = 7). Data are shown as the mean ± SEM. *p < 0.05; **p < 0.01; ***p < 0.001. BMDC, bone marrow derived dendritic cell; IFN, interferon; mtDNA, mitochondrial DNA; mtDNA (T), mtDNA transfection; Veh (T), vehicle transfection.

The mtDNA can be sensed by several pathways, such as cGAS‐STING pathway, TLR9 pathway and NLRP3 inflammasome pathway, and so forth.^27^ cGAS senses dsDNA and catalyses the generation of the secondary messenger cGAMP.^28^ As downstream of cGAS, STING pathway was markedly activated by cytoplasmic mtDNA (Figure 4A,B), thus promoting nuclear translocation of IRF3 (Figure 4C). Cytoplasmic mtDNA also up‐regulated the phosphorylation of p65, but not the phosphorylation of IRF5 (Figure 4D,E). In fact, activated STING signalling promotes TBK1 phosphorylation, which may in turn trigger NF‐κB pathway.^29^ NLRP3 inflammasome senses mtDNA via pattern‐recognition receptor in macrophages.^30^ We also tested the NLRP3 inflammasome pathway in BMDCs after mtDNA transfection, and found that cytoplasmic mtDNA did not significantly up‐regulate the NLRP3 inflammasome pathway (Figure 4F,G). Thus, we focused on whether STING signalling was required for cytoplasmic mtDNA‐mediated immunoparalysis of BMDCs.

### 3.5 STING was required for cytoplasmic mtDNA‐mediated immunoparalysis of BMDCs

**FIGURE 5:**
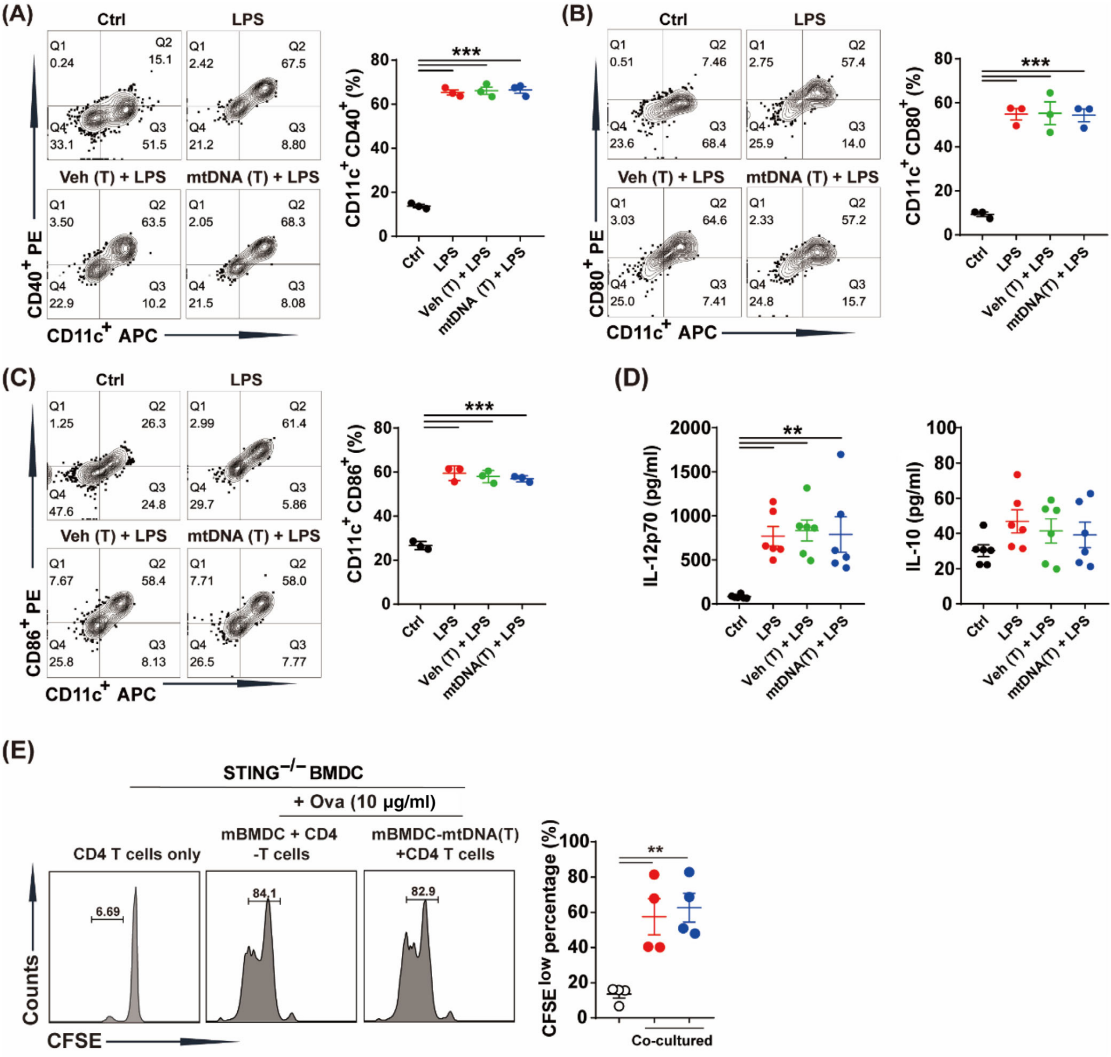
Cytoplasmic mtDNA failed to induce immunoparalysis of STING^−/−^ BMDCs. (A) Proportion of CD11c^+^CD40^+^ cells treated with LPS (100 ng/ml) or pre‐transfected with vehicle or mtDNA (10 μg/ml) for 24 h and then stimulated with LPS (100 ng/ml) for 24 h (*n* = 3). (B) Proportion of CD11c^+^CD80^+^ cells treated as (A) (*n* = 3). (C) Proportion of CD11c^+^CD86^+^ cells treated as (A) (*n* = 3). (D) IL‐12p70 and IL‐10 in cultured supernatant of STING^−/−^ BMDCs treated as (A) (*n* = 6). STING^−/−^‐BMDCs were stimulated with LPS (100 ng/ml, 24 h) to activate it (mBMDC) or pretreatment with mtDNA (T) for 24 h and stimulated with LPS (100 ng/ml, 24 h) to activate it (mBMDC‐mtDNA [T]), then incubated with Ova for 2 h, thereafter co‐cultured with CD4^+^ T cells. (E) Proliferation of CD4^+^ T cells when co‐cultured with STING^−/−^ BMDCs (*n* = 4). Data are shown as the mean ± SEM. *p < 0.05; **p < 0.01; ***p < 0.001. BMDC, bone marrow derived dendritic cell; CFSE, 5,6‐carboxyfluorescein diacetate succinimidyl ester; LPS, lipopolysaccharide; mtDNA, mitochondrial DNA; mtDNA (T), mtDNA transfection; Ova, ovalbumin; Veh (T), vehicle transfection.

STING was knockout (Figure S3) to investigate whether STING was required for cytoplasmic mtDNA‐mediated immunoparalysis of BMDCs. We then transfected STING^−/−^ BMDCs with mtDNA followed by LPS stimulation. Flow cytometry results showed that STING deficiency reversed cytoplasmic mtDNA‐induced inhibition of costimulatory molecules (CD40, CD80 and CD86) expression (Figure 5A–C). STING deficiency also promoted the proinflammatory phenotype of BMDCs after LPS challenge (up‐regulation of IL‐12p70 and down‐regulation of IL‐10) (Figure 5D). In addition, cytoplasmic mtDNA failed to inhibit the ability of STING^−/−^ BMDCs on the proliferation of CD4^+^ T cells (Figure 5E). These results indicated that STING was required for cytoplasmic mtDNA‐mediated immunoparalysis of BMDCs.

### 3.6 Cytoplasmic mtDNA regulated IL‐10 expression by STING signalling

**FIGURE 6:**
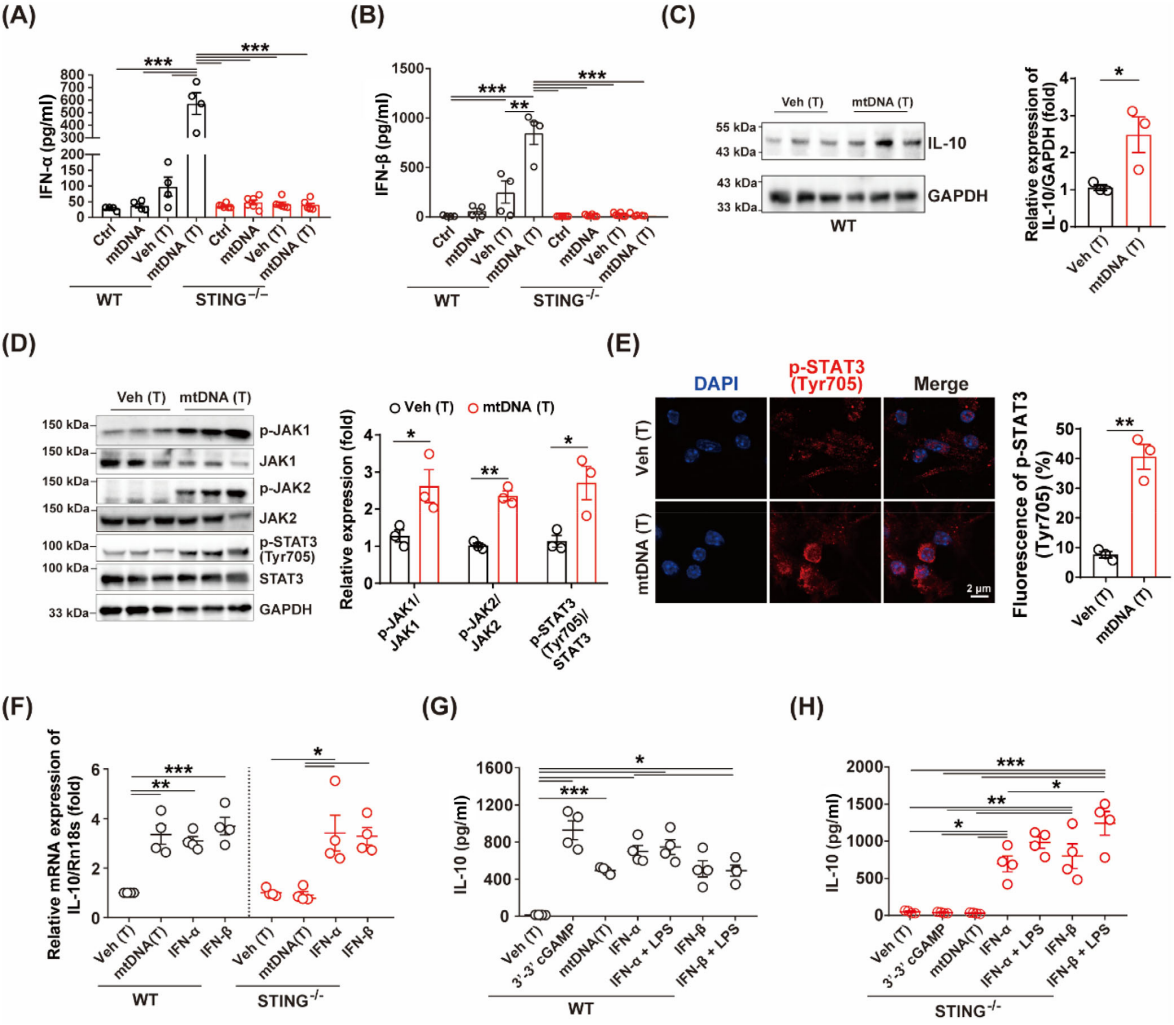
Cytoplasmic mtDNA regulated IL‐10 expression by STING‐type I IFN signalling. (A) IFN‐α in cultured supernatant of WT and STING^−/−^ BMDCs after mtDNA treatment (10 μg/ml), transfected with vehicle or mtDNA (10 μg/ml) for 24 h (*n* = 4–6). (B) IFN‐β in cultured supernatant of WT and STING^−/−^ BMDCs treated as (A) (*n* = 4–6). (C) Representative blots and relative quantification protein levels of IL‐10 of BMDCs treated with vehicle or mtDNA (10 μg/ml) for 24 h (*n* = 3). (D) Representative blots and relative quantification protein levels of JAK–STAT3 signalling of BMDCs treated as (C) (*n* = 3). (E) Representative images of p‐STAT3 fluorescence of BMDC treated as (C) (*n* = 3). Scale bar: 2 μm. (F) IL‐10 mRNA levels of BMDCs treated with mtDNA transfection (10 μg/ml), IFN‐α (100 ng/ml) or IFN‐β (100 ng/ml) of WT or STING^−/−^ BMDCs (*n* = 4). (G) IL‐10 in cultured supernatant of WT‐BMDCs treated with vehicle transfection, 3′‐3′ cGAMP (10 μg/ml), mtDNA transfection (10 μg/ml), IFN‐α (100 ng/ml), IFN‐α (100 ng/ml) plus LPS (100 ng/ml), IFN‐β (100 ng/ml) or IFN‐β (100 ng/ml) plus LPS (100 ng/ml) for 24 h (*n* = 4). (H) IL‐10 in cultured supernatant of STING^−/−^‐BMDCs treated as (G) (*n* = 4). Data are shown as the mean ± SEM. *p < 0.05; **p < 0.01; ***p < 0.001. BMDC, bone marrow derived dendritic cell; cGAMP, cyclic GMP–AMP; DAPI, 4′,6‐diamidino‐2‐phenylindole; IFN, interferon; LPS, lipopolysaccharide; mtDNA, mitochondrial DNA; mtDNA (T), mtDNA transfection; Veh (T), vehicle transfection; WT, wild type.

Cytoplasmic mtDNA promoted the release of IFN‐α/β, but not in STING deficient DCs (Figure 6A,B). It was indicated that IFN‐α was able to increase the IL‐10 levels in Behçet disease (BD, a chronic systemic inflammatory disorder) patients.^31^ In the study, cytoplasmic mtDNA also promoted the expression of IL‐10 in BMDCs (Figure 6C). IFN‐α/β was reported to bind to the type I IFN receptor (IFNAR), thus associating with JAK–STAT signalling to regulate the transcription of IFN‐stimulated genes (ISGs).^32^ It was reported that type I IFNs induced IL‐10 in a STAT3‐dependent manner.^33^ so, we detested the expression of JAK–STAT3 signalling, and found that mtDNA transfection triggered the phosphorylation of JAK‐1/2 and STAT3 (Figure 6D,E). Our data indicated that cytoplasmic mtDNA activated JAK–STAT3 signalling. Release of IL‐10 is one of the hallmarks of DCs immunoparalysis.^7^ In this study, STING deficiency was found to effectively counteract cytoplasmic mtDNA‐induced IL‐10 expression (Figure 6F–H). Collectively, these results demonstrated that cytoplasmic mtDNA regulated IL‐10 secretion via STING‐type I IFN signalling, which may be the potential mechanism by which mtDNA mediated immunoparalysis of DCs.

### 3.7 STING‐deficiency alleviated immunoparalysis of spleen DCs and sepsis‐associated inflammatory injury in vivo

**FIGURE 7:**
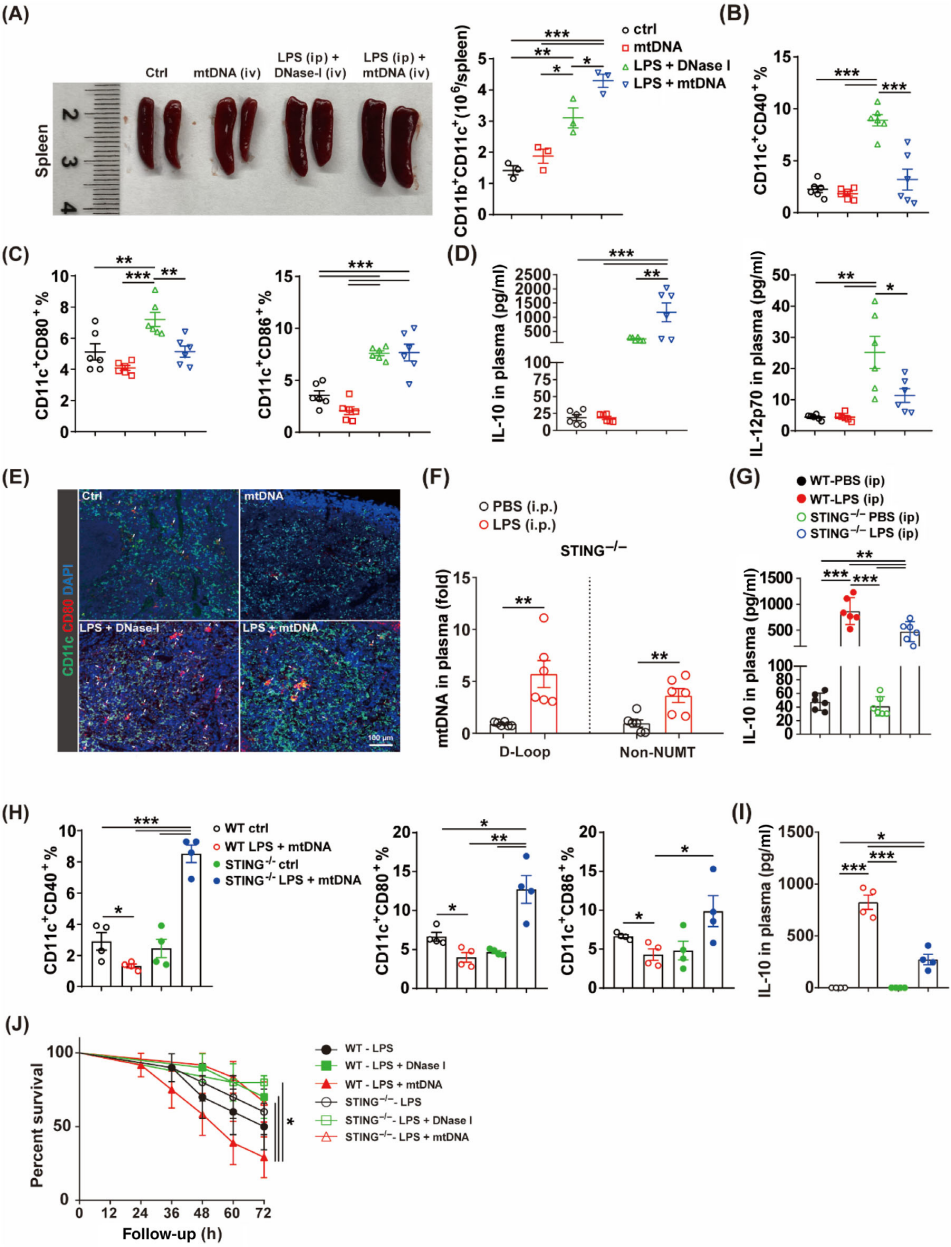
STING deficiency moderated mtDNA‐mediated immunoparalysis of spleen DCs in vivo. (A) Representative image of spleens and proportion of CD11b^+^CD11c^+^ cells of mice treated with PBS (ip, 200 μl), mtDNA (iv, 100 μg/mice), LPS (ip, 10 mg/kg) + DNase I (iv, 5 mg/kg) or LPS (ip) + mtDNA (iv, 100 μg/mice) (*n* = 4). (B) Proportion of CD11c^+^ CD40^+^ cells in spleen of mice treated as (A) (*n* = 6). (C) Proportion of CD11c^+^ CD80^+^ and CD11c^+^ CD86^+^ cells in spleen of mice treated as (A) (*n* = 6). (D) IL‐10 and IL‐12p70 in plasma of mice treated as (A) (*n* = 6). (E) Immunofluorescence staining of CD80 in spleen of mice treated as (A). Scale bar: 100 μm. (F) Plasma mtDNA levels of STING^−/−^ mice treated with PBS (ip, 200 μl) or LPS (ip, 10 mg/kg) (*n* = 6). (G) Plasma IL‐10 levels of WT and STING^−/−^ mice treated as (F) (*n* = 6). (H) Proportion of CD11c^+^ CD40^+^, CD11c^+^ CD80^+^ and CD11c^+^ CD86^+^ cells in spleen of WT and STING^−/−^ mice treated with LPS (ip, 10 mg/kg) + mtDNA (iv, 100 μg/mice) (*n* = 4). (I) Plasma IL‐10 levels of WT and STING^−/−^ mice treated as (H) (*n* = 4). (J) Survival rate of WT and STING^−/−^ mice treated with LPS (ip, 10 mg/kg) or LPS (ip, 10 mg/kg) + DNase I (5 mg/kg) or LPS (ip, 10 mg/kg) + mtDNA (iv, 100 μg/mice) (*n* = 10–12). Data are shown as the mean ± SEM or SE. *p < 0.05; **p < 0.01; ***p < 0.001. DC, dendritic cell; LPS, lipopolysaccharide; mtDNA, mitochondrial DNA; WT, wild type.

Then, we injected mtDNA via tail vein plus intraperitoneal injection of LPS in vivo. The mtDNA plus LPS led to marked splenomegaly and the increase in splenic DCs number (Figure 7A). Moreover, flow cytometry indicated that mtDNA plus LPS led to a decrease of CD40 and CD80 levels in spleen DCs, which was reversed after hydrolysing mtDNA by using DNase I (Figure 7B,C). In addition, mtDNA along with LPS promoted the release of IL‐10 and inhibited the release of IL‐12p70 (Figure 7D). Immunofluorescence staining also indicated that the expression of CD80 was inhibited in spleen DCs of mice treated with mtDNA plus LPS (Figure 7E). These results indicated that mtDNA promoted the deterioration of immunosuppressive stage in sepsis. Furthermore, we determined whether STING deficiency reversed the mtDNA‐mediated immunosuppressive state. STING^−/−^ mice treated with LPS challenge were also associated with elevated plasma mtDNA levels (Figure 7F). Notably, STING^−/−^ mice showed lower plasma IL‐10 (Figure 7G) and higher IL‐12p70 levels (Figure S4A) after LPS challenge. Septic mice with immunosuppression are vulnerable to secondary infection. Mice were treated with repeated LPS challenge (to mimic secondary infection) (Figure S4B). STING deficiency alleviated lung injury (Figure S4C,D) and improved survival rate (Figure S4E) after repeated LPS challenge. STING^−/−^ mice were also treated with tail vein injection of mtDNA plus intraperitoneal injection of LPS in vivo. The mtDNA plus LPS failed to induce significant splenomegaly, as well as increased contents of spleen DCs in STING^−/−^ mice (Figure S4F). STING deficiency reversed mtDNA‐mediated down‐regulation of CD40, CD80 and CD86 in splenic DCs (Figure 7H), inhibited the release of IL‐10 (Figure 7I) and reversed the decrease of IL‐12p70 (Figure S4G). In addition, STING deficiency improved survival rate of mice treated with mtDNA plus LPS in vivo (Figure 7J). Taken together, these data indicated that STING deficiency improved immunoparalysis of splenic DCs and prognosis of sepsis.

### 3.8 Elevation of peripheral mtDNA levels may be associated with immunosuppression in septic patients

**FIGURE 8:**
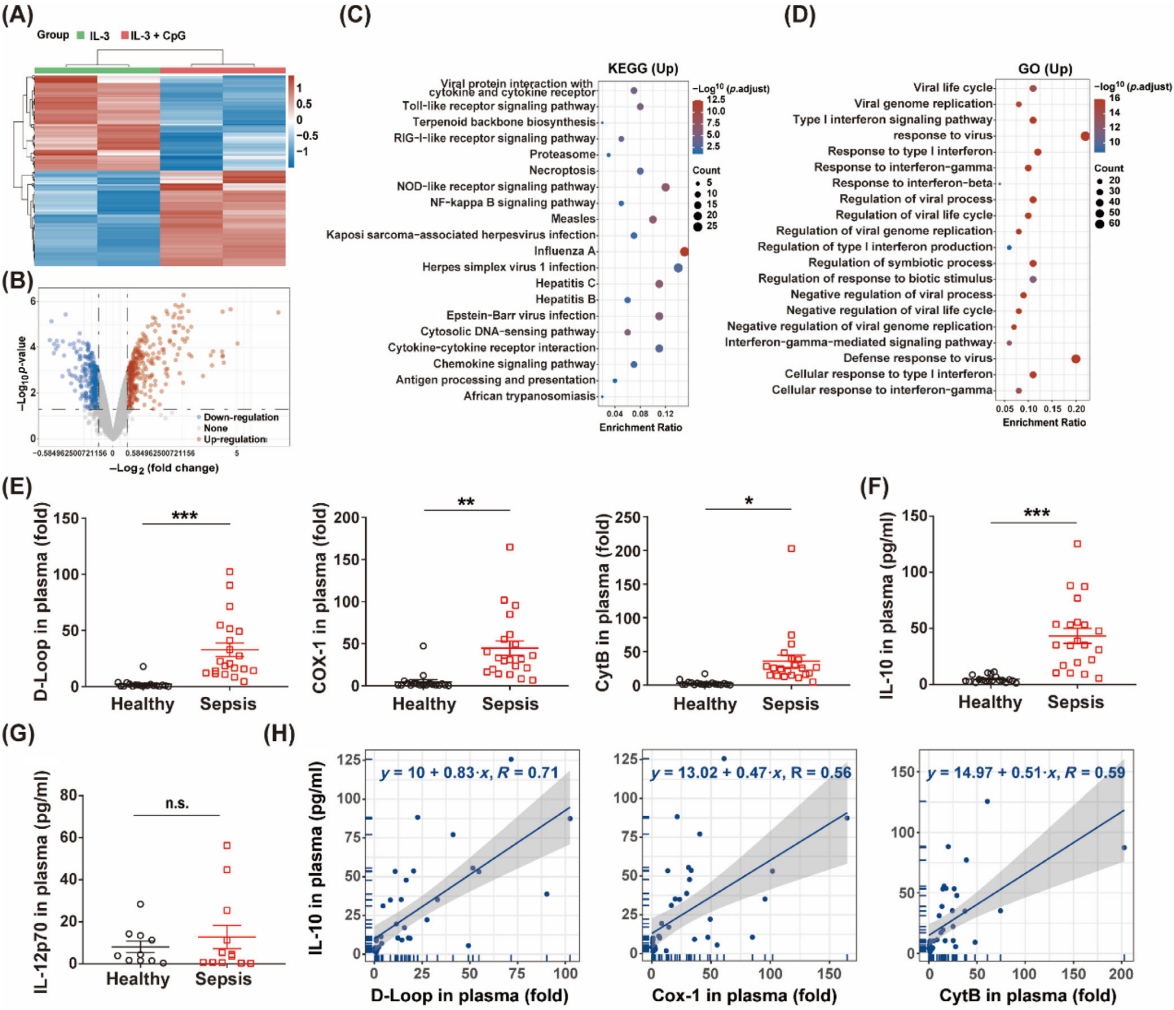
Elevated peripheral mtDNA may associate with immunosuppression of septic patients. (A) Hierarchical clustering analysis of mRNAs, which were differentially expressed between human plasmacytoid dendritic cells treated with IL‐3 or IL‐3 plus CpG (GSE10147). (B) Volcano plots were constructed using fold‐change values and adjusted *p*‐values. The red point represents the over‐expressed mRNAs and the blue point indicates the down‐expressed with statistical significance. (C) The top 20 enriched KEGG signalling pathways of up‐regulated mRNAs. (D) The top 20 GO analysis of up‐regulated mRNAs. (E) Peripheral mtDNA levels of healthy (*n* = 19) and septic patients (*n* = 21). (F) Plasma IL‐10 levels of healthy (*n* = 19) and septic patients (*n* = 21). (G) Plasma IL‐12p70 levels of healthy (*n* = 10) and septic patients (*n* = 12). (H) Correlation analysis between IL‐10 levels and plasma mtDNA (D‐Loop, Cox‐1 and CytB) levels. Data are shown as the mean ± SEM. *p < 0.05; **p < 0.01; ***p < 0.001. GO, gene ontology; GSE, gene expression omnibus series; KEGG, Kyoto Encyclopedia of Genes and Genomes; mtDNA, mitochondrial DNA.

Since mtDNA has similar structure with bacterial CpG, we selected GSE10147 from GEO database (https://www.ncbi.nlm.nih.gov/geo/). Figure 8A,B exhibits the hierarchical clustering analysis and volcano plots of the dataset. The KEGG pathways and GO functions analysis of up‐regulated differentially expressed genes. The result indicated that cytosolic DNA‐sensing pathway and regulation of type I IFN production played an important role in sepsis (Figure 8C,D). In this study, plasma samples were collected from 40 individuals (21 septic patients and 19 healthy participants). Compared with the healthy participants, septic patients were associated with higher peripheral mtDNA levels (Figure 8E), as well as IL‐10 secretion (Figure 8F), but not IL‐12p70 levels (Figure 8G). Correlation analysis showed that peripheral mtDNA levels may be related to plasma IL‐10 (Figure 8H), indicating that mtDNA may mediate immunosuppression in sepsis.

## 4 DISCUSSION

**FIGURE 9:**
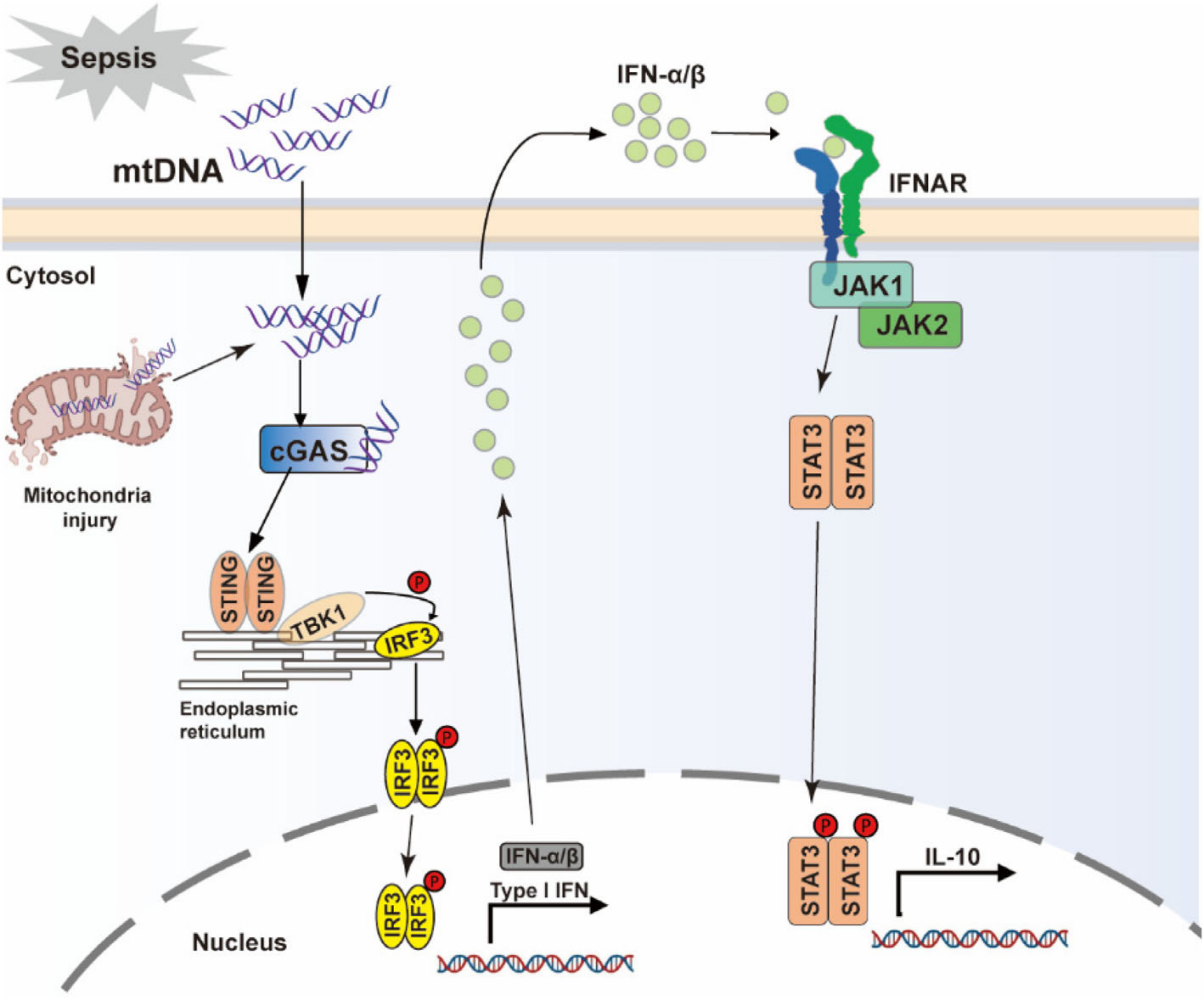
Role of mtDNA in mediating immunoparalysis of DCs. Sepsis led to mtDNA accumulation in cytoplasm of DCs. Cytoplasmic mtDNA activated STING signalling to up‐regulate phosphorylation of TBK1, promote the phosphorylation and nuclear translocation of IRF3 to mediate transcription of type I IFNs, which then triggered the JAK–STAT3 signalling to up‐regulate the transcription of IL‐10. DC, dendritic cell; IFN, interferon; mtDNA, mitochondrial DNA.

For sepsis‐induced functional alterations of DCs may modify immune responses, understanding the precise roles played by sepsis‐affected DCs is of great importance. As a potent endogenous immunostimulatory trigger, mtDNA activates inflammatory response and initiates pro‐inflammatory cascades. Previous studied indicated that elevation of peripheral mtDNA levels was associated with poor prognosis of septic patients.^11^, ^34^ However, it still remains unknown whether mtDNA is involved in immunoparalysis of DCs during sepsis. Sepsis contributes in inducing paralysed or immunosuppressive DCs, thus making it vulnerable to secondary infection.^33^ The immunoparalysis of DCs increases the levels of IL‐10 and transforming growth factor‐β, and decreases the levels of IL‐6, IL‐12p70 and costimulatory molecules, thus promoting the generation of Tregs and leading to sepsis‐associated immunosuppression.^14^ This study indicates that sepsis led to the accumulation of mtDNA in the cytoplasm of DCs. To investigate whether mtDNA contributed in inducing the immunoparalysis of DCs, mtDNA were transfected into cytoplasm of BMDCs, which significantly inhibited the expression of costimulatory molecules (CD40, CD80 and CD86) and the release of IL‐12p70, while promoting the secretion of IL‐10 in BMDCs. DCs are the most important antigen presenting cell and a link between innate and adaptive immunity. However, accumulation of cytoplasmic mtDNA inhibited the ability of DCs in promoting CD4^+^ T cell proliferation. Cytoplasmic mtDNA markedly activated the STING‐IRF3‐type I IFN pathway, and promoted the release of type I IFNs, then it triggered JAK1/2–STAT3 signalling to up‐regulate IL‐10 (a hallmark of immunosuppressive DCs), which may be the potential mechanism by which mtDNA induced immunoparalysis of DCs (Figure 9).

It has been well characterized that sepsis is associated with immune cells dysfunction and death. Hyperinflammation leads to a large amount of DAMPs accumulation, including mtDNA and HMGB1, while DAMPs may play a pivotal role in the formation of immunosuppression in sepsis.^15^ As an endogenous DAMP, mtDNA may counter‐regulate the immunologic homeostasis, switching immune activation to long‐lasting immunosuppression.^35^ Dysfunction and depletion of immune cells greatly promote the severity and persistence of sepsis‐induced immunosuppression, resulting in increased mortality to secondary infection. Therefore, understanding the mechanisms by which sepsis induced immune cells dysfunction may provide novel insights for curing sepsis. Sepsis inhibits the normal function of both splenic and lymph node DCs.^14^ In line with previous studies, mtDNA is significantly elevated in sepsis.^11^ But little is known about whether these mtDNA lead to dysfunction of DCs in sepsis.^3^ DCs were reported to uptake and sense mtDNA like macrophage, even more potent than macrophages at certain conditions.^19^ In addition, sepsis also leads to mitochondrial damage, thus mediating mtDNA accumulation in the cytoplasm. So, mtDNA was transfected into cytoplasm of BMDCs accordingly.^16^ Cytoplasmic mtDNA significantly inhibited the expression of CD40, CD80 and CD86, decreased the release of IL‐12p70 and promoted the release of IL‐10 followed by LPS challenge. Our data indicated that cytoplasmic mtDNA was involved in the formation of DCs immunoparalysis, thus tolerizing to LPS challenge.

mtDNA is reported to have immunostimulatory potentials and can directly interact with pattern recognition receptors of the innate immune system to enhance pro‐inflammatory cascades.^15^ The cGAS‐STING signalling is a crucial regulator of type I IFN in response to both exogenous and endogenous DNA. The enzyme cGAS senses cytoplasmic DNA and promotes the generation of cGAMP, which in turn binds and activates STING signalling as a second messenger, which triggers TBK1, resulting in the homodimerization and translocation to the nucleus of IRF3 to regulate the production of type I IFNs. In this study, cytoplasmic mtDNA significantly triggered STING signalling, as evidenced by phosphorylation of TBK1‐IRF3 and the promotion of type I IFNs transcription. Previous studies have indicated that mtDNA serves as an endogenous TLR9 agonist, thus involved in the development of certain disease and circulating mtDNA has a positive correlation with increased inflammatory phenotypes, as well as nuclear DNA.^36^, ^37^ However, it is difficult to determine the relative contribution of circulating nuclear DNA or mtDNA to inflammatory pathology in many cases, thus further investigations are warranted.^27^ Our results suggested that cytoplasmic mtDNA up‐regulated p65 phosphorylation. Given NF‐κB is also a downstream of TBK1, the activation of TBK1 may partially participate in the upregulation of P65 phosphorylation. It was reported that mtDNA is also regarded as an endogenous agonist of NLRP3 inflammasome, but requires the involvement of mitochondrial reactive oxygen species.^30^ In this study, mtDNA slightly promoted NLRP3 expression and IL‐1β production, but we did not detect the expression of cleavage of caspase‐1. Maybe the increase in IL‐1β secretion was achieved via TBK1‐mediated activation of NF‐κB pathway. Since cytoplasmic mtDNA markedly activated STING signalling, we mainly focused on whether cytoplasmic mtDNA mediated immunoparalysis of DCs was dependent on the STING signalling. Our results suggested that STING deficiency reversed mtDNA‐mediated immunoparalysis of DCs and moderated inflammatory injury after repeated LPS challenge. Next, we explored how STING signalling contributed in forming immunoparalysis of DCs. The release of IL‐10 is one of the hallmarks of immunoparalysis of DCs.^14^ Several reports have shown that IFN‐α increased IL‐10 levels in peripheral blood mononuclear cells of BD patients.^27^, ^31^ Like transfection of mtDNA, both IFN‐α and IFN‐β treatment promoted the production of IL‐10 in BMDCs. Type I IFN response leads to the release of IFN‐α/β which binds to IFNAR to regulate the transcription of ISGs. It was indicated that type I IFN induces the production of IL‐10 in a STAT3‐dependent manner.^33^ We also demonstrated that transfection of mtDNA activated JAK1/2–STAT3 signalling in BMDCs. In addition, our results suggested that cytoplasmic mtDNA and IFN‐α/β treatments promoted the expression of IL‐10, which was reversed by STING deficiency. The results may be the potential mechanism by which cytoplasmic mtDNA mediated immunoparalysis of DCs.

Sepsis impairs the normal function of DCs, including the expression of costimulatory molecules and pro‐inflammatory cytokines, as well as proliferation and differentiation of T cells in vivo.^5^, ^38^ In addition, we injected LPS intraperitoneally and mtDNA via tail vein in vivo. The results suggested that LPS combined with mtDNA led to inhibition of costimulatory molecules expression in splenic DCs, and associated with elevated plasma IL‐10 levels, which was reversed by hydrolysis of mtDNA by DNase I or STING deficiency. These data indicated that mtDNA‐mediated immunoparalysis of splenic DCs depended on STING signalling. Furthermore, mtDNA increased mortality of endotoxic mice, while STING knock‐out improved the prognosis of endotoxic mice. Clinically, the diagnosis of sepsis was mainly based on clinical symptoms and laboratory tests. This study demonstrated that circulating mtDNA was correlated with poor prognosis of sepsis. So, mtDNA may be regarded as a biomarker or a risk factor that leads to immunosuppression and poor prognosis of sepsis.

There are some limitations in this study. First, we transfected mtDNA into BMDCs to mimic the physiological state of DCs accordingly, which was not completely similar to the real state of DCs in sepsis, as oxidative damage also contributes to mtDNA release. Second, cytoplasmic mtDNA accumulated in DCs after LPS challenge in vivo and in vitro. The results indicated that cytoplasmic mtDNA induced immunosuppression of DCs via STING signalling, and cytoplasmic mtDNA promoted the production of IL‐10. But we did not know, whether IL‐10 in turn further inhibited the normal function of DCs. Third, only 40 clinical cases were included in this study, more clinical cases may further support the hypothesis of the study, further studies are needed to justify the results of this study. Fourth, immunoparalysis of DCs deteriorate the prognosis of sepsis. It was indicated that infusion of DCs contributes in promoting anti‐tumour effects.^39^ Likewise, infusion of normal DCs to replace the immunosuppresive DCs may improve sepsis‐associated immunosuppression and the prognosis of sepsis, which deserves further researches.

## 5 CONCLUSION

Taken together, this study indicated that mtDNA as a critical DAMP to mediate immunoparalysis of DCs promoted sepsis‐associated immunosuppression. Cytoplasmic mtDNA significantly inhibited the LPS‐induced costimulatory molecules expression and IL‐12p70 production, while increasing the secretion of IL‐10 in BMDCs. Mechanistic analysis reveals that STING signalling is required for mtDNA‐mediated immunosuppression of DCs in vivo and in vitro. In vivo depletion of STING ameliorated mtDNA‐mediated immunosuppression in DCs, and relieved inflammatory injury after repeated LPS challenge. This may provide new insights to elucidate the molecular pathogenesis of immunosuppressive DCs in sepsis, and guide future pharmacological interventions for sepsis therapy.

## AUTHOR CONTRIBUTIONS

Jinbao Li and Feng Chen designed the study. Qing Tu and Yi Li performed the experiments. Jiali Zhu and Long Guo collected the clinical data. Qing Tu, Liangfang Yao and Yun Zou drafted the manuscript. Chenchen Liu, Lu Liu and Yuan Yuan analysed the data and corrected the manuscript. All the authors read and approved the final version of the manuscript.

## FUNDING INFORMATION

This study was financially supported by grants from the National Natural Science Foundation of China (No. 81971813 and 81701943).

## CONFLICT OF INTEREST

The authors declare that the research was conducted in the absence of any commercial or financial relationships that could be construed as a potential conflict of interest.

## ETHICS STATEMENT

The studies involving human participants were reviewed and approved by Medical ethics committee of Shanghai General Hospital (No. 2019KY033). Informed consents were obtained from all the individuals or their families.

## Supporting information


**Table S1** The PCR primers.
**Table S2.** Baseline characteristics of participants.

**Figure S1** CD4^+^ T cells were isolated from the spleen of OT‐II mice and labelled with CFSE, then co‐culture of BMDCs. (A) Spleen was harvested from the OT‐II mice for isolating CD4^+^ T cells and then labelled with CFSE. (B) BMDCs were transfected with vehicle or mtDNA (10 μg/ml) for 24 h, then stimulated with LPS (100 ng/ml) for another 24 h, thereafter, incubated with Ova (10 μg/ml) for 2 h. Then, BMDCs were co‐cultured with CD4^+^ T cells (1:5) for 4 days to detect the proliferation of CD4^+^ T cells. BMDCs, bone marrow derived dendritic cells; CFSE, 5, 6‐carboxyfluorescein diacetate succinimidyl ester; LPS, lipopolysaccharide; mtDNA, mitochondrial DNA.
**Figure S2**. Cytoplasmic mtDNA level of spleen DCs in sepsis mice. Data are shown as the mean ± *SEM*. **p < 0.01; ***p < 0.001. DCs, dendritic cells; CLP, cecal ligation and puncture.
**Figure S3.** Gene identification and STING expression in vivo and vitro of STING^−/−^ mice and STING^−/−^ BMDCs. (A) Agarose gel electrophoresis of DNA isolated from tail of WT, STING^+/−^ and STING^−/−^ mice. (B,C) Western blots to confirm STING knockout. BMDCs, bone marrow derived dendritic cells; WT, wild type.
**Figure S4.** STING deficiency improved prognosis of sepsis. (A) IL‐12p70 in the plasma of WT and STING^−/−^ mice treated with PBS (ip, 200 μl) or LPS (ip, 10 mg/kg) (*n* = 6). (B) Repeated LPS challenge model of WT and STING^−/−^ mice established by intraperitoneal injection with LPS (10 mg/kg, 24 h), and then re‐injection of LPS (2 mg/kg, 6 h) was used to mimic secondary infection. Thereafter, mice were sacrificed to harvest lungs for inflammatory injury detection. (C) Representative lung histology and lung histology scores. (D) Representative MPO staining and quantification of MPO positive area (*n* = 6). (E) Survival rate of WT and STING^−/−^ mice after repeated LPS challenge (*n* = 12–14). (F) Representative image of spleen and ratio of CD11b^+^ CD11c^+^ cells of spleen (*n* = 3). (G) IL‐12p70 in the plasma of WT and STING^−/−^ mice intraperitoneal injection with LPS (10 mg/kg) plus mtDNA (i.v., 100 mg/mice) (*n* = 4). Data are shown as the mean ± SEM. *p < 0.05; **p < 0.01. LPS, lipopolysaccharide; MPO, myeloperoxidase; WT, wild type.

## Data Availability

Data and materials related to this study are available from the corresponding author on reasonable request.
