# Supplementary material for: Mitochondrial DNA mediates immunoparalysis of dendritic cells in sepsis via STING signalling
Source: Cell Prolif. 2022 Sep 15;55(12):e13328. doi: 10.1111/cpr.13328 (PMC9715356; doi:10.1111/cpr.13328)
Supplement: Supplementary file 1 — Table S1 The PCR primers. Table S2. Baseline characteristics of participants. [file CPR-55-e13328-s002.docx]

| **Supplementary table 1.** The PCR primers | | |
| --- | --- | --- |
| **Gene ID** | **Species** | **Sequence (5' to 3'）** |
| CD40 | mouse | forward: 5’-TGTCATCTGTGAAAAGGTGGTC-3’ reverse: 5’-ACTGGAGCAGCGGTGTTATG-3’ |
| CD80 | mouse | forward: 5’- ACCCCCAACATAACTGAGTCT-3’; reverse: 5’-TTCCAACCAAGAGAAGCGAGG-3’ |
| CD86 | mouse | forward: 5’- TGTTTCCGTGGAGACGCAAG-3’; reverse: 5’-TTGAGCCTTTGTAAATGGGCA-3’ |
| Tert | mouse | forward: 5’- CTAGCT CATGTGTCAAGACCCTCTT-3’; reverse: 5’-GCCAGCACGTTTCTCTCGTT-3’ |
| B2m | mouse | forward: 5’- ATGGGAAGCCGAACATACTG-3’;  reverse: 5’-CAGTCTCAGTGGGGGTGAAT-3’ |
| D-Loop | mouse | forward: 5’-AATCTACCATCCTCCGTGAAACC-3’; reverse: 5’-TCAGTTTAGCTACCCCCAAGTTTAA-3’ |
| non-NUMT | mouse | forward: 5’- CTAGAAACCCCGAAACCAAA-3’; reverse: 5’-CCAGCTATCACCAAGCTCGT-3’ |
| GAPFH | mouse | forward: 5’-AGGTCGGTGTGAACGGATTTG-3’; reverse: 5’-TGTAGACCATGTAGTTGAGGTCA-3’ |
| IFN-β | mouse | forward: 5’- CAGCTCCAAGAAAGGACGAAC-3’; reverse: 5’- GGCAGTGTAACTCTTCTGCAT-3’ |
| IL-10 | mouse | forward: 5’- GCTCTTACTGACTGGCATGAG-3’; reverse: 5’-CGCAGCTCTAGGAGCATGTG-3’ |
| Rn18s | mouse | forward: 5’- TAGAGGGACAAGTGGCGTTC-3’; reverse: 5’- CGCTGAGCCAGTCAGTGT-3’ |
| D-Loop | Human | forward: 5’-ATCAACCCTCAACTATCA-3’; reverse: 5’-ACTGTAATGTGCTATGTA-3’ |
| COX-1 | Human | forward: 5’-TCATCTGTAGGCTCATTC-3’;  reverse: 5’-GGCATCCATATAGTCACT-3’ |
| CytB | Human | forward: 5’- CCTCCAAATCACCACAGGA-3’;  reverse: 5’-TGAGTAGAGAAATGATCCGTAATA-3’ |
| b-globin | Human | forward: 5'-GTGCACCTGACTCCTGAGGAGA-3'; reverse: 5'-CCTTGATACCAACCTGCCCAG-3' |

| **Supplementary table 2.** Baseline characteristics of participants | | | |
| --- | --- | --- | --- |
| Characteristics | Septic patients  (n = 21) | Healthy  (n = 19) | *P*-value |
| Demographic variables |  |  |  |
| Age [year; median (IQR)] | 62 (53-63.5) | 38 (32, 41) | < 0.0001 |
| Females/males [n (%)] | 10/11 (52.4/47.6) | 5/14 (26.3/73.7) | 0.6903 |
| BMI [kg/m^2^, median (IQR)] | 24 (22-25) | 22 (21.75-24) | 0.2598 |
| Mean arterial pressure [mmHg, median (IQR)] | 79 (76-82) | 84 (78.5-92.5) | 0.0106 |
| Mean heart rate [beat/min,median (IQR)] | 94 (87-98) | 72 (69-78) | <0.0001 |
| Clinical characteristics |  |  |  |
| Infectious variable |  |  |  |
| Pro-calcitonin concentration [μg/L, median (IQR)] | 9 (8-10) |  |  |
| C-reactive protein concentration [g/L,median (IQR)] | 10 (8-13) |  |  |
| Leukocyte concentration [10^9^/L,median (IQR)] | 13 (10-16) |  |  |
| Severity of sepsis |  |  |  |
| SOFA [points, median (IQR)] | 3 (3-4) |  |  |
| Length of hospital stay [days,median (IQR)] | 17 (9-20) |  |  |
| 30-d survival [n (%)] | 10 (58.8) |  |  |
| Primary diagnoses [n (%)] |  |  |  |
| Gastric cancer | 3 (14.3) |  |  |
| Lung cancer | 1 (4.8) |  |  |
| Colorectal cancer | 3 (14.3) |  |  |
| Cervical cancer | 3(14.3) |  |  |
| Lung disease | 2 (9.5) |  |  |
| Ureteral calculi | 1 (4.8) |  |  |
| Pancreatitis | 1(4.8) |  |  |
| Gastrointestinal disease | 6 (28.6) |  |  |
| Multiple trauma | 1 (4.8) |  |  |
| Blood cultures [n (%)] |  |  |  |
| Gram-positive only | 4 (19) |  |  |
| Gram-negative only | 9 (42.9) |  |  |
| Mixed | 7 (33.3) |  |  |
| Negative blood cultures | 1 (4.8) |  |  |
| Note: Values are presented as mean mean ± SD or n (%). IQR, interquartile range; SOFA, Sequential Organ Failure Assessment. | | | |
