# Supplementary material for: Mitochondrial DNA mediates immunoparalysis of dendritic cells in sepsis via STING signalling
Source: Cell Prolif. 2022 Sep 15;55(12):e13328. doi: 10.1111/cpr.13328 (PMC9715356; doi:10.1111/cpr.13328)
Supplement: Supplementary file 2 — Figure S1 CD4+ T cells were isolated from the spleen of OT‐II mice and labelled with CFSE, then co‐culture of BMDCs. (A) Spleen was harvested from the OT‐II mice for isolating CD4+ T cells and then labelled with CFSE. (B) BMDCs were transfected with vehicle or mtDNA (10 μg/ml) for 24 h, then stimulated with LPS (100 ng/ml) for another 24 h, thereafter, incubated with Ova (10 μg/ml) for 2 h. Then, BMDCs were co‐cultured with CD4+ T cells (1:5) for 4 days to detect the proliferation of CD4+ T cells. BMDCs, bone marrow derived dendritic cells; CFSE, 5, 6‐carboxyfluorescein diacetate succinimidyl ester; LPS, lipopolysaccharide; mtDNA, mitochondrial DNA. Figure S2. Cytoplasmic mtDNA level of spleen DCs in sepsis mice. Data are shown as the mean ± SEM. **p < 0.01; ***p < 0.001. DCs, dendritic cells; CLP, cecal ligation and puncture. Figure S3. Gene identification and STING expression in vivo and vitro of STING−/− mice and STING−/− BMDCs. (A) Agarose gel electrophoresis of DNA isolated from tail of WT, STING+/− and STING−/− mice. (B,C) Western blots to confirm STING knockout. BMDCs, bone marrow derived dendritic cells; WT, wild type. Figure S4. STING deficiency improved prognosis of sepsis. (A) IL‐12p70 in the plasma of WT and STING−/− mice treated with PBS (ip, 200 μl) or LPS (ip, 10 mg/kg) (n = 6). (B) Repeated LPS challenge model of WT and STING−/− mice established by intraperitoneal injection with LPS (10 mg/kg, 24 h), and then re‐injection of LPS (2 mg/kg, 6 h) was used to mimic secondary infection. Thereafter, mice were sacrificed to harvest lungs for inflammatory injury detection. (C) Representative lung histology and lung histology scores. (D) Representative MPO staining and quantification of MPO positive area (n = 6). (E) Survival rate of WT and STING−/− mice after repeated LPS challenge (n = 12–14). (F) Representative image of spleen and ratio of CD11b+ CD11c+ cells of spleen (n = 3). (G) IL‐12p70 in the plasma of WT and STING−/− mice intraperitoneal injection wi [file CPR-55-e13328-s001.docx]

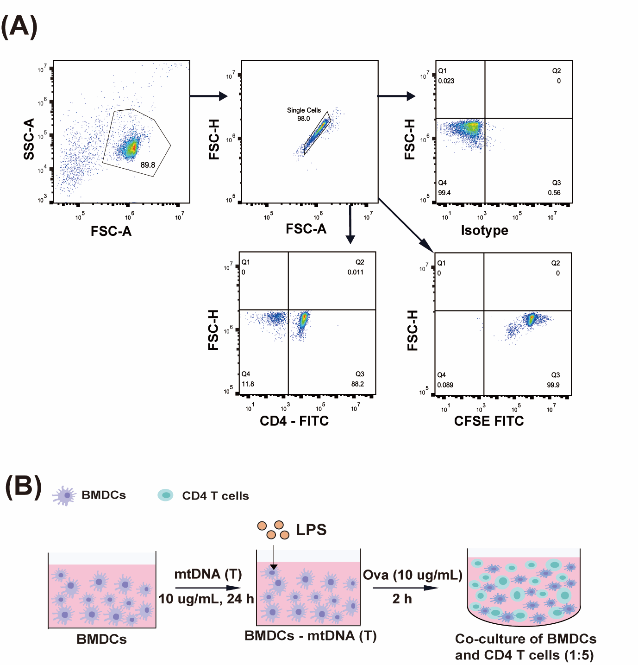


**Supplementary Figure S1.** CD4^+^ T cells were isolated from the spleen of OT-II mice and labelled with CFSE, then co-culture of BMDCs. (A) Spleen was harvested from the OT-II mice for isolating CD4^+^ T cells and then labelled with CFSE. (B) BMDCs were transfected with vehicle or mtDNA (10 ug/mL) for 24h, then stimulated with LPS (100 ng/mL) for another 24h, thereafter, incubated with Ova (10 ug/mL) for 2 h. Then, BMDCs were co-cultured with CD4^+^ T cells (1:5) for 4 days to detect the proliferation of CD4^+^ T cells. BMDCs, Bone marrow derived dendritic cells; LPS, lipopolysaccharide; CFSE, 5, 6-carboxyfluorescein diacetate succinimidyl ester.


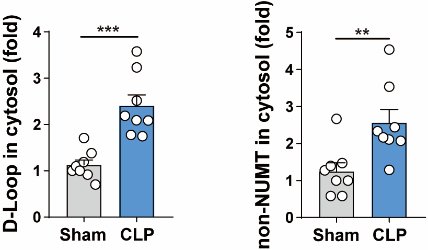


**Supplemental Figure S2**. Cytoplasmic mtDNA level of spleen DCs in sepsis mice. Data are shown as the mean ± SEM. ^**^*P* < 0.01; ^***^*P* < 0.001. DCs, dendritic cells; CLP, cecal ligation and puncture.


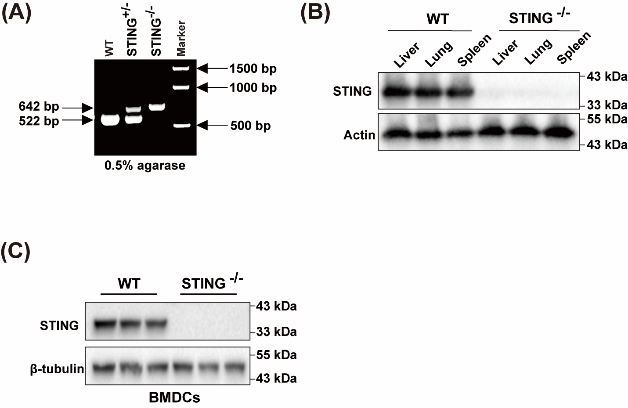


**Supplemental Figure S3.** Gene identification and STING expression in *vivo* and *vitro* of STING^-/-^ mice and STING^-/-^ BMDCs*.* (A) Agarose gel electrophoresis of DNA isolated from tail of WT, STING^+/-^ and STING^-/-^ mice. (B-C) Western blots to confirm STING knockout. BMDCs, Bone marrow derived dendritic cells; WT, wild type.


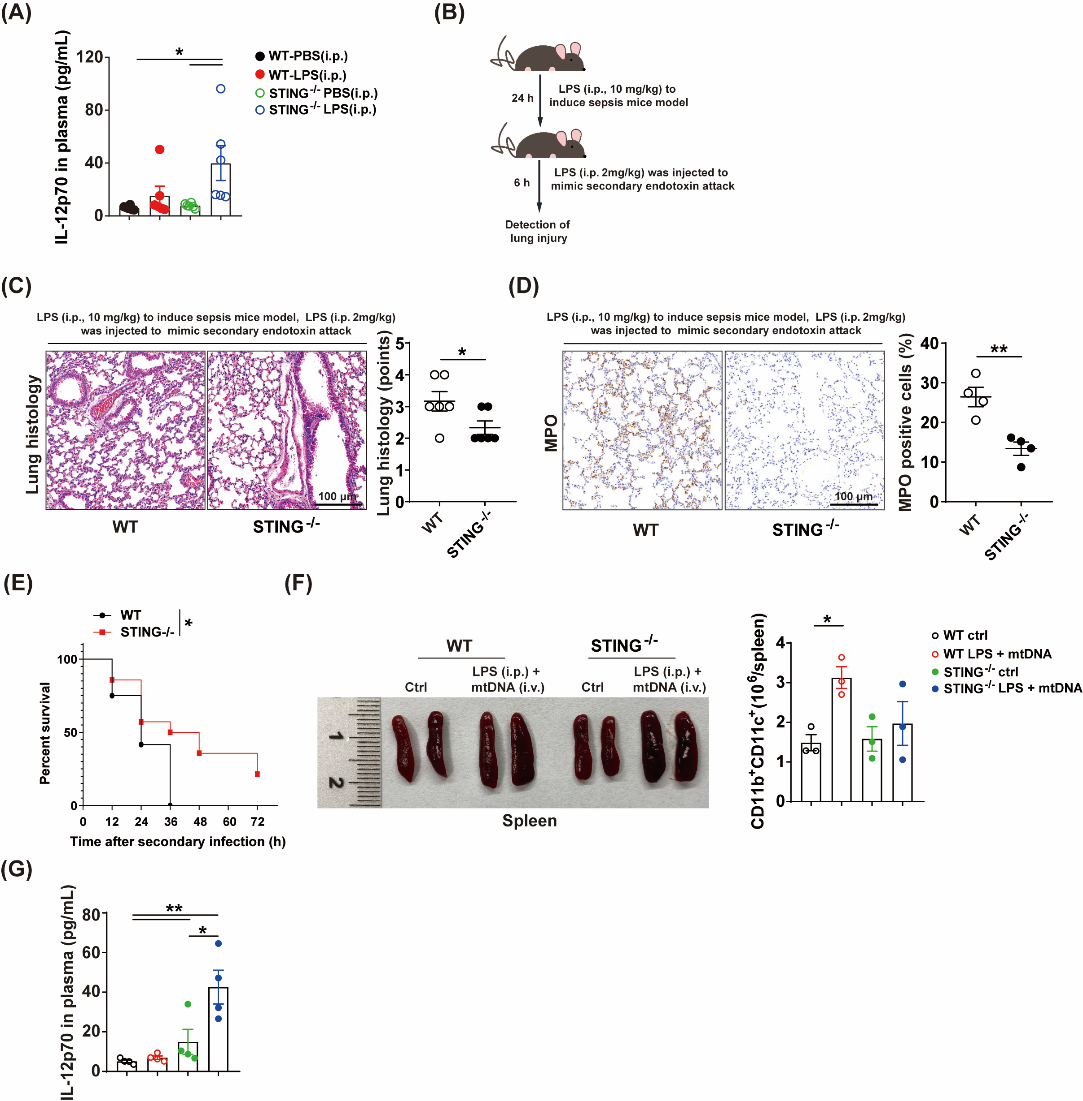


**Supplemental Figure S4.** STING deficiency improved prognosis of sepsis. (A) IL-12p70 in the plasma of WT and STING^-/-^ mice treated with PBS (i.p., 200 ul) or LPS (i.p., 10 mg/kg) (n = 6). (B) Repeated LPS challenge model of WT and STING^-/-^ mice established by intraperitoneal injection with LPS (10 mg/kg, 24 h), and then re-injection of LPS (2 mg/kg, 6 h) was used to mimic secondary infection. Thereafter, mice were sacrificed to harvest lungs for inflammatory injury detection. (C) Representative lung histology and lung histology scores. (D) Representative MPO staining and quantification of MPO positive area (n = 6). (E) Survival rate of WT and STING^-/-^ mice after repeated LPS challenge (n = 12-14). (F) Representative image of spleen and ratio of CD11b^+^ CD11c^+^ cells of spleen (n = 3). (G) IL-12p70 in the plasma of WT and STING^-/-^ mice intraperitoneal injection with LPS (10 mg/kg) plus mtDNA (i.v., 100 mg/mice) (n = 4). Data are shown as the mean ± SEM. ^*^*P* < 0.05; ^**^*P* < 0.01. WT, wild type; LPS, lipopolysaccharide; MPO, myeloperoxidase.
